# Virtual reality in stroke recovery: a meta-review of systematic reviews

**DOI:** 10.1186/s42234-024-00150-9

**Published:** 2024-10-05

**Authors:** Ammar Khan, Yahia Z. Imam, Mohamed Muneer, Salman Al Jerdi, Sumanjit K. Gill

**Affiliations:** 1 Weill Cornell Medicine in Qatar, PO Box 24144, Doha, Qatar; 2 Hamad Medical Corporation, PO Box 3050, Doha, Qatar; 3 UCL Institute of Neurology, Queen Square, London, UK

**Keywords:** Virtual reality, Stroke recovery, Rehabilitation, Systematic reviews

## Abstract

**Background:**

Virtual Reality (VR) is an emerging technology in post stroke recovery. However, its precise role in stroke rehabilitation is not well defined. The aim of this paper is to conduct an overview of systematic reviews on the role of VR in stroke rehabilitation.

**Methods:**

A meta-review with results from a search of 7 databases from inception till 5^th^ December 2022 with subsequent quality appraisal was conducted. The primary outcome was to produce a narrative review on the efficacy of VR versus usual or other care in stroke recovery. Data was synthesized in a descriptive fashion and high-quality systematic reviews were emphasized. The AMSTAR-2 tool was used for quality assessment of the included studies.

**Results:**

Evidence from high-quality systematic reviews suggests that there is benefit from VR in upper limb, lower limb, gait, and balance recovery particularly when additive to conventional therapy. There is also limited evidence to suggest that VR has a positive effect in those with impaired cognition.

**Conclusion:**

VR is safe and effective as an adjunct to conventional therapy for adults after stroke and should be used routinely for upper and lower limb motor recovery. Further high-quality studies that evaluate its efficacy and explore ways to increase its positive impact in areas such as cognition are required. There is also a scope for the development of stroke-specific virtual environments. (PROSPERO registration # CRD42022372926).

**Supplementary Information:**

The online version contains supplementary material available at 10.1186/s42234-024-00150-9.

## Background

Stroke is the major cause of mortality and disability in the world affecting over 17 million people annually (Uthman 2016; Aminov et al. 2018; Feigin et al. 2017). While advancements in medical technology and treatments have led to a decrease in stroke mortality and incidence in high-income countries, patients continue to suffer from long-term neurological deficits, including cognitive, behavioral, functional, language, and mobility deficits (Aminov et al. 2018).

Stroke rehabilitation is a complex process which optimizes recovery of injured neural tissue through enhancement of neural repair, maximizing recovery and minimizing functional deficits (Homberg 2013; McDowell 1994; Krucoff et al. 2016). The mainstay of stroke rehabilitation is a combination of physical, occupational, speech and cognitive psychological therapy, requiring multi-disciplinary input (Teasell et al. 2009). To be effective, stroke rehabilitation should include goal oriented, task-specific training (Perez-Marcos et al. 2017), sufficient duration and intensity of the intervention i.e. high repetitive volume (Lang et al. 2015) and utilization of biofeedback (Langhorne et al. 2009). This can be challenging in terms of costs (Jutai and Teasell 2003), time constraints (Bayley et al. 2012) and in keeping the patients engaged and motivated (Laut et al. 2015).

Virtual Reality (VR) is theorized to overcome these limitations particularly those of cost and time constraints. VR is defined as “a computer rendered, 3-dimensional, real-time, interactive experience of artificial reality containing items, characters, and events existing only in the memory of a computer” (Stasieńko and Sarzyńska-Długosz 2016).

The user is provided with visual feedback either on a head mounted device, a computer monitor, or a screen of any type and can interact with the virtual environment (VE) through multiple mechanisms. The platform used to interact with VR is termed an environment; and the environment can be immersive, semi-immersive, or non-immersive. Immersive environments are where the subject is surrounded by the virtual environment providing a high degree of realism and immersiveness. This can be achieved through the use of head-mounted devices. A semi-immersive environment is one with a moderate level of realism and immersion, falling in between a fully immersive and a non-immersive environment. A non-immersive environment in one where subjects are fully responsive to the real environment and the virtual environment is viewed via the use of high-resolution monitors and computer devices.

Key concepts in the use of VR are immersion, imagination, and interaction (Hao et al. 2022a). Immersion is the extent to which the user perceives that they are in the virtual environment rather than the real world (Gaggioli 2009). The rapid increase and development of video game technology has made semi immersive or non-immersive VR cost effective and available for use in clinical practice (Proffitt and Lange 2015). These commercial gaming platforms simulate real life situations and require total body movement similar to the real world and encourage high intensity repetitive hand movements such as seen with the Nintendo Wii and PlayStation gaming platforms (Thomson et al. 2014; Casserly and Baer 2014). Immersive environments are thought to be superior due to increased levels of user engagement, participation, and enjoyment (Hao et al. 2022a; Moan et al. 2021). These environments, however, are not in routine use yet due to lack of guidelines for their use in stroke rehabilitation as well as being more expensive and sophisticated to use.

### Virtual reality in stroke rehabilitation

Earlier studies that used functional imaging showed that functional improvement is associated with ipsilesional activation of the sensorimotor cortex post VR training in patients post stroke (Laver et al. 2018). This has continued to drive forward and support the use of VR for stroke rehabilitation.

The functional recovery of damaged brain tissue is heavily driven by neural plasticity (Hao et al. 2022a). Neural plasticity is the ability of the central nervous system to adapt and undergo dynamic changes in terms of structural and functional components in response to experiences and feedback received through the different senses (Hao et al. 2022a). The underlying neural mechanisms of this adaptability and change are dependent on the strength of the synaptic connections and axonal remodeling of the cortical pathways (Dimyan and Cohen 2011). To effectively target neural plasticity and functional recovery through rehabilitation, the rehabilitation technique needs to involve goal oriented, intensive, repetitive, and task-specific measures that are reiterated by constant visual and sensory feedback to the user from the environment (Hao et al. 2022a). VR seems to be able to meet these criteria for efficacy based on functional imaging of patients post-stroke (Laver et al. 2017).

The mirror neurons are a class of visuomotor neurons involving interconnected brain regions (premotor cortex, inferior parietal lobule, and inferior frontal gyrus) that play a role in processing information related to the execution of movements (Hao et al. 2022a). Imitation and imagery have been seen to activate some of the regions of this mirror neuron system in the past (Hao et al. 2022a). Since the VR environment depicts the user as an avatar on a screen, this means that the patients can also see themselves performing the task through the avatar, much like standing in front of the mirror. Therefore, when that same avatar performs a motion, the mirror neurons in the brain can then be activated allowing the user to initiate that specific motion.

VR, when originally developed, was thought to have the potential to revolutionize stroke rehabilitation by providing the flexibility of outpatient treatment as well as by increasing patient engagement, satisfaction, and enjoyment. This remains true to this day. Enjoyability (Tussyadiah et al. 2018) is believed to be one of the main attractive features of VR. This enjoyment may improve motivation to practice and allows for more therapy time (Corbetta et al. 2015). This is further enhanced by the sense of presence (Immersion) (McMahan 2003), feeling of success or accomplishment (Joseph et al. 2014), and synchrony (Tarr et al. 2018) (playing with other participants and/or engaging in competition). Immersion level is a significant factor that can affect a user’s enjoyment, engagement, and response level. Studies have shown that more immersion leads to an increased sense of a user’s presence in the virtual environment, better learning experience and retrieval movement for virtual objects in post-stroke patients (Hao et al. 2022a). A more immersive, enriched, interactive, multi-component environment with multimodal stimulation can allow the user to do more complicated tasks and has also been shown to significantly affect both patients’ and clinicians’ engagement, participation, and satisfaction (Moan et al. 2021).

Earlier studies included a smaller number of participants (Jack et al. 2001; Kim et al. 2012) and utilized a different set of outcome measures, which made drawing conclusions and systematically reviewing these studies, a difficult task. Nonetheless, these studies have shown promising results. Meta-analyses have suggested some benefit of VR systems in improving motor function after stroke (Laver et al. 2015; Saposnik and Levin 2011). A review looking at the effect of specific over non-specific VR-based rehabilitation (NSVR) on post-stroke recovery (Maier et al. 2019) concluded that specific VR-based rehabilitation was more beneficial in improving Upper Limb (UL) recovery than Conventional Therapy (CT), however, non-specific VR was not. This study showed immensely promising results along with highlighting certain principles of VR-based rehabilitation that explain why VR is superior to CT for post-stroke patients. However, the conclusions put forward by this study require further investigations since the number of studies included in the NSVR category was relatively small and may have contributed to the low statistical power of the study. Moreover, the reviews involved were heterogenous in terms of the outcomes measured, time after stroke, and dosage or frequency of the intervention. More recently, several systematic reviews and meta-analyses have been published comparing VR to CT assessing the improvement in UL function, lower limb (LL) function, balance, gait, cognition, and aphasia. These studies were heterogeneous to an extent in terms of the type of VR intervention used, the outcomes measured, and the conclusions drawn (Wiley et al. 2022; Parisi et al. 2022). Due to the large amount of preliminary data with inconclusive, yet promising results, it is important to compile the current systematic reviews to gather current evidence in order to pave the way for virtual reality to be more routinely accessible to patients suffering from post-stroke deficits with clear and evidence-based guidelines for its use.

Therefore, this overview of systematic reviews aims at studying VR in a larger context, critiquing available systematic reviews and summarizing in a descriptive manner the available evidence to conclude whether VR is superior to the conventional rehabilitation therapy post stroke across different functional domains. It is hoped that our qualitative analysis, if favorable for VR, would pave the way for establishing guidelines for the routine use of VR either in combination with CT or alone in improving recovery post-stroke.

## Methods

This is an overview of systematic reviews (A meta-review of systematic reviews).

### Review question

What is the effectiveness of VR in comparison to conventional rehabilitation or no care in stroke recovery?

### Searches strategy

Synonyms of VR and stroke were used and adapted to different databases and searched using Boolean operators (AND/OR). The detailed search syntax is available in Appendix 1. Published manuscripts from inception up to 5^th^ December 2022 were identified by using electronic and manual searches of the Cochrane Database of Systematic Reviews, the Database of Abstracts of Reviews of Effectiveness, PsycINFO, EMBASE, Physiotherapy Evidence Database (PEDro), Web of Science and Medline in December 2022. Search limits (English, Humans, Systematic Reviews, Meta-analysis) were employed to select articles. Relevant reference lists of identified studies and published reviews were manually checked for additional reviews. The results of the electronic search were examined for duplicate entries using the ‘find duplicates’ facility of reference management software (EndNote X8) and were manually crosschecked.

Studies with mixed etiology groups were excluded unless participants’ stroke-specific data was available.

### Participants/population

This review includes all systematic reviews on studies that have applied VR for rehabilitation of patients after stroke targeting various outcomes including aphasia, motor, neglect, cognition, executive function, and gait recovery.

#### Inclusion criteria

1. Adults above 18 years of age.
2. Post stroke (ischemic or hemorrhagic, any time).
3. Systematic reviews.
4. Only English text will be included.
5. Peer reviewed and published.
6. Therapy including a form of VR as a key part of the therapy provided.
7. Therapy targeting language function, motor function, cognitive, executive function, or neglect.
8. Report impairment and/or activity and/or participation-oriented outcome measures.

#### Exclusion criteria

1. Therapy that does not include a form of VR.
2. Therapy that included exogenous stimulation (such as robotic aid or functional electrical stimulation).
3. Subjects who were animals or children.
4. Reviews were excluded if they were not systematic, i.e., did not have a formal method section detailing how selection bias was excluded.
5. Non-English text

### Intervention

This review considered systematic reviews that included studies that applied VR, immersive or non-immersive, for rehabilitation (language, cognition, motor, gait, neglect, and functionality) after stroke on its own or in addition to usual care.

### Control

Usual care, conventional rehabilitation, any other forms of exercise, or no treatment.

### Primary outcome

Upper limb function, lower limb function, balance, gait, global cognition, language, memory, attention, visuospatial awareness evidenced from the included high-quality studies.

### Data extraction

The obtained search results were first screened using the title and abstract utilizing the inclusion/exclusion criteria. Full texts were then analyzed for quality and content. Reporting was according to the Preferred Reporting Items for Systematic Reviews and Meta-Analyses (PRISMA) statement framework for reporting of systematic reviews (Moher et al. 2009). Two assessors independently reviewed the search process against the inclusion and exclusion criteria, and the risk of bias assessment. Disagreements were discussed until consensus was achieved. Data was then extracted by the two independent authors and summarized as in Table S1 of the supplementary file.

### Quality assessment of included studies

The AMSTAR-2 (Shea et al. 2017) (Appendix 2) tool was used for quality assessment of the included studies due to its ease of use, accessibility, and broad assessment of the quality of systematic reviews AMSTAR 2 contains 16 items, and is used to rate reviews as high, moderate, low, or critically low quality depending on the presence of critical or non-critical weaknesses. Critical weaknesses are defined by Shea et al. 2017 in appendix 2.

### Review registration

Before the search was initiated, the protocol was drafted and was registered with PROSPERO. The unique registration number is CRD42022372926.

### Strategy for data synthesis

A narrative (descriptive) synthesis was conducted utilizing the evidence, results, and conclusions drawn from the high and moderate-quality reviews only. However, wherever applicable, results from low-quality reviews were also analyzed to reinforce or contradict the conclusions drawn from the high and moderate quality reviews and this was specifically mentioned as such in the results section. A quantitative synthesis including a meta-analysis could not be done due to the amount of heterogeneity and diversity present in these reviews in terms of outcome measures.

Outcomes were grouped according to how they were presented in the reviews. The major outcomes were grouped as upper limb outcomes, lower limb outcomes, gait and balance outcomes, and cognition outcomes. Additionally, several moderators and factors that could influence these outcomes were also analyzed and these included degree of immersion, virtual reality platform, time since stroke, and dosing of the intervention.

### Evidence map

A visual map of the evidence from each systematic review or article was created (Fig. 2) to visually display the conclusions of each review and included 4 dimensions as per the map created by Miake-Lye et al. (2019).

1. Number of original studies (bubble size): The number of studies included in each review is represented proportionally by the size of the bubble.
2. Outcome measured (bubble color): The outcome of the review (UL rehabilitation, LL rehabilitation, or cognition) will be determined from each bubble’s color.
3. Effect (x-axis): The authors classified each review according to the effects found and conclusions drawn. When the interventional group showed greater benefits than the control group, the intervention was classified as “better”; otherwise, the intervention was classified as “worse”. When there was insufficient evidence or if a specific conclusion could not be drawn, the intervention was classified as “mixed/unclear”. If there were no differences, the intervention was included as “no differences”.
4. Strength of evidence (y-axis): The reviews were sorted into the following 4 categories high strength of evidence, moderate strength of evidence, low strength of evidence, or very low strength of evidence. This grading depended on several factors such as the specific article’s recommendation, their findings, their assessment of the evidence, and effect size. The AMSTAR-2 ratings of the articles did not affect this grading. If each article explicitly provided its level of recommendation, this was represented. If it was not explicitly reported, this was inferred by the authors of this article.

## Results

### Description of the included systematic reviews

**Fig. 1:**
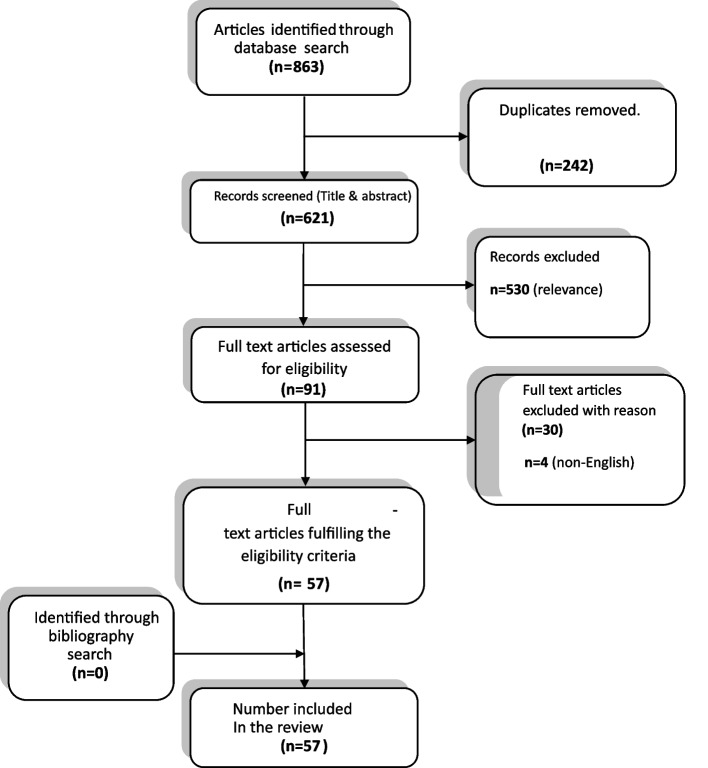
The selection process summarized in the PRISMA diagram

A search of various databases, including Medline, PEDro, Cochrane Database of Systematic Reviews, Database of Abstracts of Reviews of Effectiveness, EMBASE, PsycINFO, and Web of Science, yielded a total of 863 references. After eliminating duplicate references and applying inclusion and exclusion criteria at both the title and abstract level and at the full text level, 57 references met the criteria for inclusion. A detailed list of excluded full text articles can be found in Appendix 3. Upon reviewing the reference lists of the included reviews, no additional references were identified. Thus, a total of 57 systematic reviews were included in this study. The selection process adhered to the PRISMA flow diagram (Moher et al. 2009), and a summary of this process can be seen in Fig. 1.

Our overview included a total of 57 articles: 15 were systematic reviews and 42 were systematic reviews with meta-analysis. These articles were published between 2007 and 2022. The number of participants per review ranged from 47 to 3,540. The articles included 1,033 randomized controlled trials (RCTs) and 152 non-randomized or observational studies. Most of the recent systematic reviews (2015–2022) reported selectively on RCTs, while earlier reviews also included observational and non-randomized studies. See table S1 for details.

Most of the reviews included studies utilizing virtual environments (VE) and commercial gaming (CG) platforms. Only 15 systematic reviews (Crosbie et al. 2007; Henderson et al. 2007; Smith et al. 2012; Cavalcanti Moreira et al. 2013) investigated only VE; whereas, only 7 (Thomson et al. 2014; Cheok et al. 2015; Dos Santos et al. 2015) investigated CGs alone.

In 46/57 reviews VR was either added to CT and compared to CT alone or was compared to CT alone without the combination and in 11 it was compared to CT alone or no therapy.

The reviews reported various measurements outcomes; these include 14 on upper limb recovery (Casserly and Baer 2014; Saposnik and Levin 2011; Henderson et al. 2007), 5 on balance recovery (Chen et al. 2016; Li et al. 2016), 5 on gait recovery (Cavalcanti Moreira et al. 2013; Rodrigues-Baroni et al. 2014), 7 on lower limb recovery including gait and balance (Imam and Jarus 2014; Luque-Moreno et al. 2015), 3 on cognition alone (Parisi et al. 2022; Moher et al. 2009; Fernández-Vázquez et al. 2022) and the rest reporting on different combinations of the above.

### Assessment of methodological quality of included systematic reviews

Table S2 shows the AMSTAR 2 grade (high, moderate, or low) confidence in the result with missing critical domains noted. Appendix 4 shows the individual reviews’ detailed AMSTAR 2 item scoring. Only 13 reviews were noted to be of methodological good quality (moderate to high) according to the AMSTAR 2 tool, these are (Aminov et al. 2018; Parisi et al. 2022; Smith et al. 2012; Lohse et al. 2014; de Rooij et al.. 2016; Iruthayarajah et al. 2017; Cao et al. 2021; Doumas et al. 2021; Cortes-Perez et al. 2021; Garay-Sánchez et al. 2021; Zhang et al. 2021a; Chen et al. 2022a); 15 were critically low with multiple critical domains missing, and the remaining 28 were of low methodological quality (Table S2 and Appendix 4).

The most common missing domain relates to questions 2 and 7 (writing and registering a protocol with the relevant search information and providing a list of excluded full text articles respectively) and question 10 (reporting on the sources of funding for the included studies). 16 reviews had a prior registration; 15 with PROSPERO (Li et al. 2016; Lohse et al. 2014) and one with the Cochrane database (Laver et al. 2018). The search strategy was judged to be comprehensive in over 60% of the reviews while the rest were partially comprehensive. 50/57 reviews explicitly disclosed conflicts of interest and/or funding sources, none of the reviews explicitly discussed funding of the studies included in their respective reviews (Question 10 on the AMSTAR 2 tool).

### Extracted outcome measures

Outcomes from the high and moderate quality reviews were extracted and summarized, as the confidence in these reviews’ results was moderate to high.

### Upper limb outcome

The major results and conclusions drawn from the high-moderate quality studies have been summarized in the supplementary table S1. Most of these studies used the Fugl Meyer assessment (FMA) to evaluate the effects of VR on UL function. Consensus from the 8 high to moderate quality reviews show that VR enhances UL recovery, particularly if additive to CT allowing more therapy time. The effect size was moderate at best. Laver et al. (2018) have shown an improvement in activities of daily living (ADL) when compared to CT but not UL recovery, whereas others (Aminov et al. 2018; Smith et al. 2012; Lohse et al. 2014; Doumas et al. 2021; Cortes-Perez et al. 2021) have shown improvement of UL structure and function. Doumas et al. (2021) used leap motion controller, a form of semi-immersive VR that consists of a device with sensors designed to detect, recognize, and capture hand gestures and finger positions in addition to generating a virtual image of the UL on a screen indicating the user the next task to be performed. This device was found to be more effective than CT in improving grips strength (low-quality evidence, medium-high effect) and UL-mobility-oriented tasks (large effect, low-quality evidence). Chen et al. (2022b) showed statistically significant improvement in UL motor function, muscle strength, range of motion and independence in day-to-day activities. The UL motor function was measured using the Fugl Meyer assessment (FMA), Manual Muscle Testing (MMT), Motricity Index (MI) and several other scales. Independence in day-to-day activities was measured using scales such as Functional Independence Measure (FIM), and Barthel Index or modified Barthel Index. This review also showed that when VR rehabilitation exercises were combined with CT, this led to improvement in hand dexterity. Fernández-Vázquez et al. (2022) showed the same effect as Chen et al. (2022b) when VR was combined with CT. The evidence, however, is conflicting with regards to the use of VR versus CG. Laver et al. (2018) have shown a trend toward rehab-specific VR to be more beneficial and that CG is not superior when compared to CT, whereas Lohse et al. (2014) have shown effectiveness for both VR and CG. Other reviews (Saposnik and Levin 2011; Thomson et al. 2014; Dos Santos et al. 2015) agree with Lohse et al. (2014), however these reviews are observed to be of lower methodological quality.

Reviews (Fernández-Vázquez et al. 2022; Doumas et al. 2021; Cortes-Perez et al. 2021; Chen et al. 2022b) looked at UL rehabilitation post stroke and all of them concluded that there was statistically significant improvement in UL motor function with the use of VR either in combination with CT or alone. Fernández-Vázquez et al. (2022) has shown that the combination of haptic gloves, semi-immersive VR, and CT produce significant improvement in UL functionality (measured by the FMA), Jebson-Taylor Hand Function Test (JTT), or the Block and Box test (BBT) as compared to CT alone.

Apart from these high to moderate quality reviews, the low and critically low-quality studies have also shown similar results (Table S1).

### Lower limb recovery, gait and balance outcomes

Most systematic reviews have included outcomes related to lower limbs, balance and walking together as they are interlinked. Several scales were used commonly throughout these studies to measure LLand balance. For example, for balance and gait, Brunel Balance Assessment (BBA), Berg Balance Scale (BBS), Dynamic Gait Index (DGI), Fugl-Meyer Assessment balance subscale, Postural Assessment Scale for Stroke and Balance Evaluation Systems Test were some of the scales used in these reviews to measure the LL functionality.

Two reviews (Smith et al. 2012; de Rooij et al. 2016) demonstrated benefit of VR when added to CT on gait and balance, one (Lohse et al. 2014) demonstrated benefit across all three outcomes.

Other reviews focusing specifically on balance recovery (Chen et al. 2016; Li et al. 2016) have shown improvement in static and dynamic balance or improvement in balance in timed up and go test. A recent review (Garay-Sánchez et al. 2021) evaluating the effect of VR on static and dynamic balance showed significant improvement in static balance when non-immersive VR was used in combination with CT, whereas for dynamic balance, 2 of the reviews in the systematic review showed improvement with immersive VR and 4 reviews showed significant improvement with non-immersive VR both in combination with CT. Likewise systematic reviews focused solely on gait recovery have shown VR to improve walking speed (Rodrigues-Baroni et al. 2014) and distance walked (Cavalcanti Moreira et al. 2013) but the confidence in results of these reviews remain low due to heterogeneity between studies, lower number of participants involved, and lack of blinding of therapists and participants.

### Cognitive outcomes

Four high quality reviews (Aminov et al. 2018; Parisi et al. 2022; Lohse et al. 2014; Zhang et al. 2021a) (reporting on 7 RCTs) included a cognitive component in their VR assessments. The cognitive domains tested were memory, neglect/visual training, and executive function. The high-quality reviews (Aminov et al. 2018; Lohse et al. 2014) showed positive effects of VR (used either in combination or without CT) on cognition with a small to medium effect size. The high-quality review (Parisi et al. 2022) showed that multisensory technology that includes VR both with and without motion tracking but more so the former, is more effective than conventional therapy for cognition especially for specific domains such as attention, visuospatial processing, memory, and global cognition. There was one high quality review (Cao et al. 2021) that looked at functional communication as the main outcome of VR (immersive and semi-immersive), however, it was concluded that was no significant difference between VR and the control group in the review. There was also considerable heterogeneity in the results of the reviews included. A high-quality review by Zhang et al. (2021a) assessing global cognition as well as domain-specific outcomes such as attention, executive function, memory, and verbal fluency revealed no significant effect on global cognition with the use of VR but improved effects on executive function, memory, and visuospatial function. Another high-quality review by Wiley et al. (2022) published recently looked at multi-sensory technology and its effects on cognition, language, executive function, and memory post-stroke. The review found that multisensory technologies without motion tracking were more effective than standard therapies in improving the mentioned domains whilst multi-sensory technology with motion tracking was similar to the conventional group 3 weeks after the interventions.

Overall, VR had a positive effect on cognition, effect size was noted to be modest, and the studies were noted to be heterogenous.

### Moderators of outcome

#### Degree of immersion

Most studies included reviews with varying degrees of immersion (immersive, semi-immersive and non-immersive), however a few reviews reported on the impact of immersion on outcome.

Henderson et al. (2007) found immersive virtual reality (IVR) to be beneficial when compared to no therapy, the authors did not find any studies at the time of conduction of the review on IVR versus CT. They also found NIVR to be less effective than IVR versus no therapy, but outcomes failed to reach significance when compared to CT. A high-quality review by Smith et al. (2012) focusing primarily on NIVR found it to be useful as an adjunct to CT but there was little evidence to suggest improvement of outcomes when it was compared to CT alone. Another high-quality review grouped the interventions into 2 groups (immersive and non-immersive VR) and looked at the effects on static and dynamic balance (Garay-Sánchez et al. 2021). Four studies using NIVRwithin this review showed improvements in static balance whilst a single study using immersive VR showed the same. For dynamic balance, there were 4 non-immersive VR and 2 immersive VR studies that showed favorable outcomes. The low number of studies using immersive VR in the field of neurological disorders can be attributed to the scarce usage of immersive VR devices due to their high cost and availability. However, the results do show promising effects comparable to non-immersive VR. A review (Fernández-Vázquez et al. 2022) looked at the combination of haptic gloves (greater interaction between the user and the object with more feedback) with semi-immersive VR and their effects on UL motor rehabilitation. This review showed that this combination resulted in significant improvement in UL functionality as compared to CT alone.

Overall, there is evidence to suggest that immersive VR is as effective as non-immersive or semi-immersive VR in improving functional outcomes post-stroke. The recent pilot review published looking at fully immersive VR and evaluating patient and clinician’s perceptions showed that patients experienced a greater deal of motivation, felt more engaged, and experienced more enjoyment than what would have been possible in CT (Tarr et al. 2018). Theoretically, this would lead to better outcomes and increased compliance to the rehabilitation. This shows that there is a great deal of potential in implementing fully immersive VR in post-stroke rehabilitation, however, more evidence is needed to clearly study the effects of immersion of improvement in post-stroke deficits.

#### Virtual reality platform

The 3 reviews investigating CG as a VR platform reported varying degrees of improvement in the ADL, UL outcomes and static balance. However, these reviews have emphasized CG as an adjunct rather than a replacement of CT. Among the high quality reviews, (Aminov et al. 2018) found rehab-specific VE platforms to be superior to CG, (Laver et al. 2018) found a trend favoring VR over CG and that CG were not more beneficial than CT in UL recovery. Lohse et al. (2014) while demonstrating that both VR and CG are beneficial stated that “current CG interventions have been too few and too small to assess potential benefits of CG”.

Recent studies have used more CG devices and semi-immersive VR such as Nintendo Wii and Xbox Kinect since these are now readily available and at a lower cost. Serious games are also being used in certain studies (Doumas et al. 2021). A serious game is defined as a game that has education or rehabilitation as its primary goal (Doumas et al. 2021). These games would use motion capture systems, robotic exoskeletons, or a simple smartphone or tablet computer. It was seen that rehabilitation through serious games led to better improvements in motor function, activity, and participation as compared to CT. According to (Cortes-Perez et al. 2021), leap motion controller video games have also been shown to improve UL function post-stroke especially when combined with CT. A combination of haptic glove systems combined with CG devices and semi-immersive VR also produces statistical improvement in the outcomes measured. Other low-quality studies have also demonstrated possible benefits of including CG and gaming devices in stroke rehabilitation (Khan et al. 2023; Peng et al. 2021; Chan et al. 2022). A review (Chan et al. 2022) looked solely at exergaming (video games that require people to interact with the thorough purposeful body movements) at improving functional outcomes in patients with chronic stroke and it was found that exergaming showed statistically significant improvement in balance, lower limb functional mobility, and functional independence (Chan et al. 2022).

#### Time since stroke

The overwhelming majority of participants were in their chronic phase of stroke (> 6 months), however some reviews included patients in the acute (1 month) and subacute (1–3 months) phase post stroke. Aminov et al. (2018) found no significant differences between overall outcome in patients receiving VR therapy at the subacute and the chronic phases of their stroke. Lohse et al. (2014) could not draw conclusions as the trials were small in size with not enough statistical power for regression analysis. Laver et al. (2017) found no statistically significant difference between stroke patients recruited within 6 months after stroke to those recruited after 6 months. Chan et al. (2022) found that patients with subacute stroke found greater improvements in arm and hand motor ability than those with chronic stroke. However, patients with chronic stroke showed greater improvements in quality of life than patients with subacute stroke. Parisi et al. (2022) used multi-sensory technology with and without motion tracking and found that the group with patients in the subacute stroke stage (3–6 months) benefited the most from the intervention. Fernández-Vázquez et al. (2022) concluded that in the very acute (< 1) month stage, the use of haptic gloves and semi-immersive VR was superior to conventional treatment in the UL functionality regardless of whether it was combined with CT or not. However, for the long-term improvement in UL functionality, the significant effects of the haptic gloves and semi-immersive VR were only preserved if they were combined with CT.

Hence, there is evidence to suggest rehabilitation is more effective in the subacute and acute stages of stroke than the chronic stage, however, outcomes such as quality of life improve greatly when rehabilitation is done more than 6 months after stroke.

### Dosing intervention

VR interventions were delivered in variable ways with respect to intensity, frequency, and duration of the intervention. Laver et al. (2018) compared trials applying under 15 h of intervention with trials applying 15 h or more of intervention on upper limb function and found no significant difference. Aminov et al. (2018) found no significant difference for different doses, durations, and frequencies of VR intervention. Study (Chen et al. 2022b) by Chen et al. showed that “receiving > 15 h of VR intervention (SMD 0.92, 95% CI 0.35–1.49; *P* = 0.002) was associated with significant improvements in hand dexterity (BBT) compared with receiving ≤ 15 h of VR intervention (SMD − 0.10, 95% CI − 0.35 to 0.15; *P* = 0.45)” (Chen et al. 2022b). Also, “receiving VR-supported exercise therapy for > 1 month (SMD 0.97, 95% CI 0.06–1.89; *P* = 0.04) was associated with greater improvements in hand dexterity (BBT) than receiving VR-supported exercise therapy for < 1 month” (SMD 0.02, 95% CI − 0.22 to 0.26; *P* = 0.84). However, those who received trial lengths of 2 weeks to 1 month (SMD 0.49, 95% CI − 0.11 to 1.10; *P* = 0.11) showed greater improvements in quality of life than those for whom trial lengths were > 1 month (SMD − 0.20, 95% CI − 0.46 to 0.06; *P* = 0.13)” (Chan et al. 2022). Study (Laver et al. 2017) by Laver et al. concluded that at least 15 h of rehabilitation was needed to achieve significant improvements in UL functionality, however in review (Fernández-Vázquez et al. 2022), there were 2 studies that showed significant improvement in UL functionality with having done less than 15 h. This was most likely due to the higher intensity of VR applied in these studies (5 sessions per week in consecutive days). Therefore, the differences can be attributed to the duration as well as the intensity of VR rehabilitation.

### Adverse effects

**Fig. 2:**
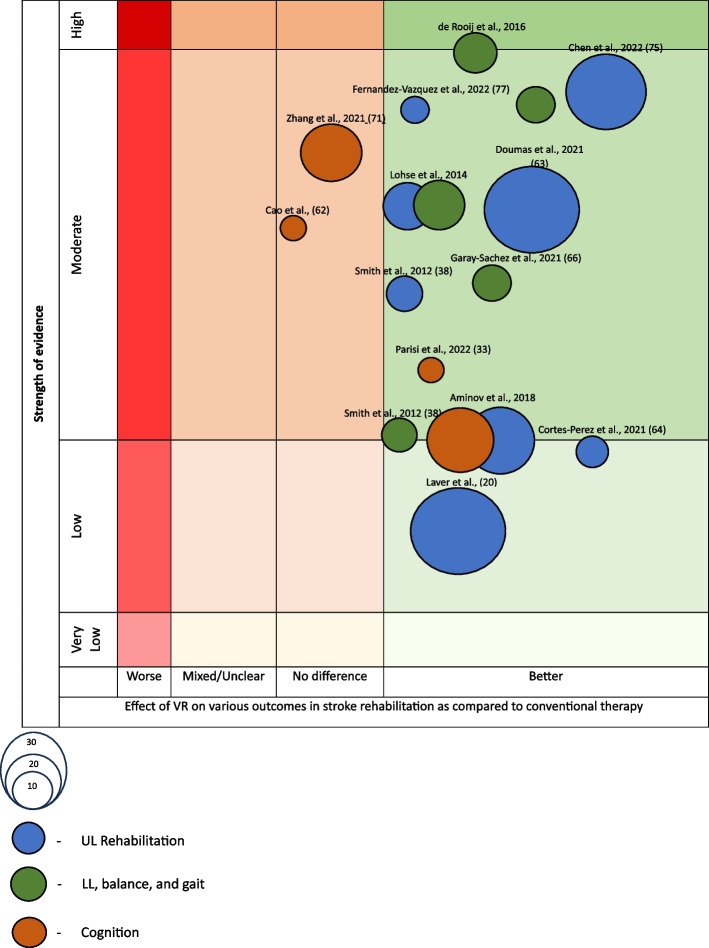
Evidence map of the effect of VR on various post-stroke outcomes in different high and moderate quality studies

The reviewed systematic reviews seldom mention adverse events. However, when these were reported, they were found to be infrequent and mild in nature. These include, headache, dizziness, pain and increased tone (Laver et al. 2018) (Fig. 2).

This figure has been created by the author and does not require permission to be included in the article.

## Discussion

This overview of systematic reviews on the effect of VR on stroke recovery aims at synthesizing and summarizing available evidence from multiple systematic reviews. This allows evidence to be consolidated and recommendations to be strengthened and made easily accessible to clinicians. Additionally, in areas of research where evidence is thin or non-existent, this overview has helped uncover these pertinent areas and generate questions for future research to fill gaps in the current body of literature. Finally, we have used the AMSTAR-2 tool to evaluate the articles for several reasons including its ease of use and accessibility. Moreover, AMSTAR-2 provides a broad assessment of the quality, including flaws that may have arisen through poor conduct of the reviews. It also identifies critical and non-critical domains which has helped the authors stratify the articles into high-medium, and low-quality reviews.

### Virtual reality is beneficial and safe

Evidence from multiple high-quality reviews incorporating high-quality RCTs suggests that VR improves upper limb recovery, balance, gait, and cognition post stroke when added to conventional therapy above and beyond conventional therapy alone. The effect is postulated to be by providing further therapy time, however there seems to be an effect even when therapy is time-matched (Aminov et al. 2018). Furthermore, it is shown to be safe with rare and mild side effects.

### Virtual reality effect on UL mobility, LL mobility, balance, gait

Almost all the high to moderate quality reviews as well as the low-quality reviews concluded that VR rehabilitation produced statistically significant improvement in UL and LL function, more specifically gross motor function. Thus, from this review we have sufficient good quality evidence to support the claim that VR use should be encouraged for patients with post-stroke rehabilitation. The more immersive and enriched the experience of VR is, the higher the intrinsic motivation and the higher the adherence to the therapy leading to better outcomes (Hao et al. 2022a; Moan et al. 2021). There are several reasons why VR improves UL and LL motor function more than CT. VR provides access to therapeutic exercises in an environment that stimulates real life experiences and interaction which otherwise the patients may not have been able to access (Chen et al. 2022b). Moreover, VR provides real-time feedback to the user through various senses including sounds and vibration sense. Positive feedback encourages and motivates users to continue and engage in the therapy, something that CT is unable to provide. Thirdly, VR also provides intensive, goal-oriented, and repetitive tasks involved in exercises that promote muscle coordination and neuronal development. With continued engagement in therapy, the connections among neurons can be strengthened and reorganization of the regions in the cerebral cortex corresponding to the affected extremity can also be induced, utilizing the concept of neural plasticity as mentioned previously (Chen et al. 2022b). Additionally, VR games have built in reward systems for achieving and reaching certain milestones and this further encourages and motivates users to continue the therapy (Chen et al. 2022b).

In one study, patients suffering from UL deficits were asked to reach targets appearing in a virtual environment using a virtual avatar over several trials. In some of these trials, the researchers amplified the movement of the paretic limb’s virtual representation, making it appear faster, more accurate, and easier to reach the target on the screen. These manipulations were suppressed gradually, and participants were kept unaware of them. It was noted that after such amplification, patients started using their paretic limb more frequently and only 10 min of enhancement was enough to induce significant changes in the amount of spontaneous use of the paretic limb. Thus, changing a patient’s belief in their capabilities can improve deficits post-stroke. This also suggests the importance of the involvement the of mirror neuron system in virtual reality systems (Ballester et al. 2015).

The same concepts can be applied to the improvement seen in lower limbs, thus improving gait and balance. When a patient is asked to walk in a virtual environment, the environment can be manipulated to include specific obstacles and constraints requiring the use of problem-solving skills, which is known to be useful in enhancing the cognitive learning of new skills. This combined with a reward system, introducing positive feedback and increased virtual immersiveness, can stimulate significant improvement in gait and balance.

The high-quality study (Cortes-Perez et al. 2021) done by Cortes-Perez et al., focused on leap motion controller (LMC), a VR haptic body recognition device, equipped with sensors and cameras that tracks the movements of the arm, wrist, and hand. It is cheap, small, and fairly accurate at body recognition and tracking. According to this study, LMC was shown to be effective in improving upper limb mobility in patients with stroke, especially when combined with CT. It requires continuous and repetitive interactions, thus favoring brain plasticity. Moreover, since it allows high precision gesture recognition, it allows for training of the UL with games that mimic or include ADLs such as cooking, dressing, eating, etc.

### Virtual reality effect on cognition

It is important to note that there is a scarcity of studies on cognition and the few studies done contain considerable amount of heterogeneity in their outcomes.

The current data from the available reviews supports the use of VR in post stroke cognitive impairments. This is supported by evidence from other neurological disciplines (Hofmann et al. 2003; Davidsdottir et al. 2008) demonstrating that VR can be used for cognitive re-training and could be a valid option were CT has shown shortcomings such as anosognosia (Joseph et al. 2014).

Recently, several systematic reviews have been published incorporating cognition as one of the outcomes of VR rehabilitation. Out of the 4 high-quality reviews, Zhang et al. (2021a) concluded that there was no significant improvement in terms of global cognition, however, there was significant improvement seen in executive function, memory, and visuospatial function post stroke. One of the reasons for the conflicting results on cognition may be due to the fact that the VR exercises and rehabilitation interventions may not be focused solely on improving cognition, rather they target other outcomes such as UL and LL function whilst looking at cognition as a secondary outcome. It remains unclear whether increasing VR therapy focusing just on cognition would lead to significant improvement.

One explanation of the impact of virtual reality (VR) on cognition is the cognitive rehabilitation theory, which explains that intense and repeated sensory stimulation and functional training can enable the brain areas surrounding damaged tissue to compensate for the lost functions of the damaged regions (Chen et al. 2022a). Moreover, some reviews have shown that VR stimulates improvement in the excitability of the remaining neurons, improves functional reorganization of the damaged brain area, and forms new neural circuits (Chen et al. 2022a). Other studies have stated that VR rehabilitation “activates brain metabolism, increases cerebral blood flow, and the release of neurotransmitters” (Zhang et al. 2021a), thus leading to improved cognitive function.

Nonetheless, more studies looking specifically at this population of patients with post-stroke cognitive impairment are required utilizing high-quality large sample randomized controlled trials along with adequate follow-up for at least 12 months post-stroke in order to generate high quality evidence regarding the role of VR in improving cognition.

### Influence of moderators on outcomes

#### Time since stroke

Most of the reviews included patient in the chronic stage of stroke (> 6 month) with only a few of the included trials including patients in the acute and subacute phases.

Although a high-quality review reporting on high quality evidence (Aminov et al. 2018) found no statistical difference between VR applied and acute/subacute stage of recovery, as both were equally effective, however optimal timing to apply VR remains to be explored further (Aminov et al. 2018). Another high quality study (Lohse et al. 2014) looked at time post-stroke for all the outcomes measures as a potential confounding factor but concluded that it did not influence any of the outcomes measured.

Previous research has shown that the majority of gains and motor recovery occur within the first 1–3 months post-stroke (Cassidy and Cramer 2017). So, capitalizing on this “window of opportunity” of peaked neuroplasticity in the initial period after stroke makes sense biologically (Chen et al. 2002; Wang et al. 2017).

However, there is some evidence that application of VR in the subacute phase (3–6 month) may be more beneficial. Wang et al. (2017) have shown that applying Leap Motion VR which can track the fine movements of both hands and fingers in the subacute phase of stroke is feasible and promising. Another study (Parisi et al. 2022) used multi-sensory technology with and without motion tracking also found that the group with patients in the subacute stroke stage benefited the most from the intervention.

Additionally, Chan et al. (2022) found that patients with subacute stroke found greater improvements in arm and hand motor ability after being subjected to VR and exergaming interventions than those with chronic stroke. Patients with chronic stroke showed greater improvements in quality of life after VR rehabilitation and exergaming than patients with subacute stroke (Chan et al. 2022). This is most likely because outcomes such as cognition, activities of daily living, and mental health all play a role in the quality of life post stroke and the recovery of cognition is dissimilar to motor recovery as it usually takes longer (Cassidy and Cramer 2017). To uncover the impact on cognition with an understanding of any nuanced effect it may have on domain specific recovery, longer follow up periods are needed, something that is currently lacking (Aminov et al. 2018). This needs to be addressed in specifically designed RCT with adequate follow-up periods after intervention.

#### Intensity, frequency and dosing of VR intervention

VR intervention in the trials included in these reviews were conducted with varying doses of intervention (intensity, frequency and duration) (Aminov et al. 2018). Some trials did not report the dosing of the intervention, and when dosing was provided, true dose- matching between interventional and control arms, was not ensured (e.g., “matching active time in therapy or numbers of repetitions”) (Aminov et al. 2018). However, there was one review that looked at the effects of VR and time dosed matched CT and it concluded that VR is superior to time-dose matched CT in terms of recovery of upper extremity motor function in patients poststroke, especially when VR is combined with CT (Li et al. 2022).

A study showed that receiving > 15 h of VR intervention was associated with significant improvements in hand dexterity (BBT) compared with receiving ≤ 15 h of VR intervention (Chen et al. 2022b). Also, receiving VR-supported exercise therapy for > 1 month was associated with greater improvements in hand dexterity (BBT) than receiving VR-supported exercise therapy for < 1 month. However, those who received trial lengths of 2 weeks to 1 month showed greater improvements in quality of life than those for whom trial lengths were > 1 month (Chen et al. 2022b). Additionally, Laver et al. (2017) concluded that at least 15 h of rehabilitation was needed to achieve significant improvements in UL functionality, however in the review by Mohammadi et al. (2019), there were 2 studies that showed significant improvement in UL functionality with having done less than 15 sessions. This was most likely due to the higher intensity of VR applied in these studies (5 sessions per week in consecutive days). Therefore, the dosing intensity and frequency affect the various outcome measures in different ways. A longer duration of intervention may not always be more beneficial than a shorter duration for all the outcomes as seen by review (Chen et al. 2022b).

Future studies with true matching of intensity, frequency and dosing of the VR intervention are needed to help understand the benefits of VR therapy.

#### Stroke- specific virtual environment

While CGs are not typically designed for rehabilitation purposes, yet a lot of therapists tend to use them as they are available and cheap (Saposnik and Levin 2011). Although, high quality studies such as (Lohse et al. 2014) concluded that there was no evidence for differences between VE and CG games, this may also have been due to the scarce number of studies looking at CG interventions. VR effect was observed to be more robust when utilizing rehab specific VEs, however CG interventions were valuable as an adjunct to CT, but strong recommendation regarding the preferred platform for VR delivery is yet to be made. Building on the results of this review, a genuine need arises for studies on CG and for the development and testing of stroke specific VEs. Furthermore, availability and affordability of VEs is another concern since the CGs such as Nintendo Wii and Xbox Kinect are very widely available and cheap and therefore can be more routinely used in rehabilitation. Exergaming (video games that require people to interact with the thorough purposeful body movements) was found to show statistically significant improvement in balance, lower limb functional mobility, and functional independence (Chan et al. 2022). This may also partly be due to the rewarding experience inducing high intrinsic motivation leading to better adherence to the therapy.

One high quality study (Doumas et al. 2021) utilized games that are specifically developed for rehabilitation (serious games) and these showed favorable outcomes not just on UL motor function, activity, and participation but also in maintaining long term effect retention regarding UL motor function. This shows that interventions (CG, VR) that are specifically designed using neurorehabilitation principles and based on elements that enhance neural plasticity can lead to significantly better results when compared with CT alone.

## Limitations

Selection bias was unavoidable as this review included only reviews in the English language. The risk of bias cannot be ruled out as there was considerable heterogeneity even between high quality reviews pertaining to different outcomes. Most of the reviews here were of low-quality AMSTAR 2 ratings with only a handful of high-quality reviews driving the results. Furthermore, the reported evidence grading from some of the high-quality reviews was less than excellent. Additionally, a meta-analysis could not be done due to the vast number of studies and the heterogeneity within them, therefore the absence of quantitative analysis is a limitation that could be worked on in future studies.

## Conclusions

Virtual reality is a promising technology that can add to our rehabilitation armamentarium and aid recovery of the post stroke patient. This overview demonstrates that VR is a safe and effective adjunct to conventional therapy for post stroke recovery across different functional domains. It is especially beneficial when used in combination with CT. There is clear evidence supporting the use of VR rehabilitation (including exergaming and CG) in combination with CT or alone for the use of post-stroke UL and LL impairment. There is potential evidence to apply VR for cognition and balance as well, however due to the heterogeneity and conflicting results, more studies are needed to study the effects of VR on these outcomes. For now, there are clinical implications that can be derived from this meta-review and that is to routinely use VR rehabilitation (VE, CG, exergaming) for patients with post-stroke UL and LL impairment. Having said this, the heterogeneity of the studies and discrepancy in some of the outcomes has raised further questions regarding optimal dose, frequency, timing, and choice of VR intervention which should be continued to be studied in prospective well-designed clinical trials.

## Supplementary Information

Supplementary Material 1: Table S1. Summary and characteristics of the systematic reviews. Table describing all the systematic reviews discussed in this meta-review.

Supplementary Material 2: Table S2. Quality ratings of the systematic reviews using the AMSTAR-2 tool.

Supplementary Material 3.

Supplementary Material 4.

Supplementary Material 5.

Supplementary Material 6.

## Data Availability

All data generated or analyzed during this study are included in this published article [and its supplementary information files].

## Abbreviations

VR: Virtual Reality
NSVR: Non-Specific VR-based Rehabilitation
UL: Upper Limb
CT: Conventional Therapy
LL: Lower Limb
PRISMA: Preferred Reporting Items for Systematic Reviews and Meta-Analyses
RCTs: Randomized Controlled Trials
VE: Virtual Environment
CG: Commercial Gaming
FMA: Fugl Meyer Assessment
ADL: Activities of Daily Living
MMT: Manual Muscle Testing
MI: Motricity Index
FIM: Functional Independence Measure
JTT: Jebson-Taylor Hand Function Test
BBT: Block and Box Test
BBA: Brunel Balance Assessment
BBS: Berg Balance Scale
DGI: Dynamic Gait Index
NIVR: Non-immersive Virtual Reality
IVR: Immersive Virtual Reality
SMD: Standardized mean difference
CI: Confidence interval
MMSE: Mini-Mental State Examination
MoCA: Montreal Cognitive Assessment
LOTCA: Loewenstein Occupational Therapy Cognitive Assessment
RBMT-II: Rivermead Behavioral Memory Test Second Edition
