# Supplementary material for: Virtual reality in stroke recovery: a meta-review of systematic reviews
Source: Bioelectron Med. 2024 Oct 5;10:23. doi: 10.1186/s42234-024-00150-9 (PMC11452980; doi:10.1186/s42234-024-00150-9)
Supplement: Supplementary file 1 — Supplementary Material 1: Table S1. Summary and characteristics of the systematic reviews. Table describing all the systematic reviews discussed in this meta-review. [file 42234_2024_150_MOESM1_ESM.docx]

Table S1. Summary and characteristics of the systematic reviews included

| **Author** | **Date of search** | **No**  **of**  **RCTs** | **No of Non-randomized studies** | **Participants** | **Time since stroke** | **Type of**  **VR** | **Control** | **Target impairments** | **Results/**  **conclusion** |
| --- | --- | --- | --- | --- | --- | --- | --- | --- | --- |
| **Crosbie et al., 2007**  **(39)** | Inception/  1980 to February 2005 | 3 | 8 | 144 | 6 weeks-6 years | VE | No therapy or CT | Gait balance, cognition  UL and LL | Overall safe and beneficial, but not a strong recommendation |
| **Henderson et al., 2007**  **(40)** | Varies according to database from Jan 1982-Jan 2006 | 2 | 4 | 79 VR participants (no mention of controls) | 3 studies acute & 3 chronic | VE | No therapy or CT | UL | IVR Vs no therapy is beneficial  -no studies IVR VS CT  -less strong evidence NIVR Vs no therapy is effective but not Vs CT |
| **Saposnik and Levin, 2011**  **(32)** | 1966 to July 2010 | 5 | 7 | 195 | 3 subacute, 9 chronic | 9 VE;3 CG:  (4=IVR  8= NIVR) | Additive to CT or robotic OR Vs CT or no therapy | UL | 11/12 in favor of VR |
| **Smith et al., 2012**  **(41)** | Inception to December 2009 | 8 | 0 | 189 | Mostly chronic (up to 66 months) | VE  (NIRV) | Additive to CT or Vs CT or no therapy | Majority UL (5), balance and gait | VR improves UL and walking particularly if additive  but not balance |
| **Cavalcanti Moreira et al., 2013**  **(42)** | 1966 to 2011 | 4 | 0 | 72 | Any time after stroke (not specified) | VE | CT, robotic and no therapy | Gait | Better gait velocity and distance walked |
| **Casserly and Baer, 2014**  **(19)** | 1995 to 2011 | 2 | 6 | 135 | Acute, subacute & chronic stroke | VE+  GC | CT or alternative | UL | VR had a positive effect on the UL. (Evidence is of low quality) |
| **Thomson et al., 2014**  **(18)** | Inception to January 2013 | 3 | 16 | 215 | Mostly Chronic | GC | CT or no therapy | UL | CG a positive impact on ADL (3/4 +ve, UL (3/9) +ve and movement (6/11) (weak evidence) |
| **Imam and Jarus, 2014 (48)** | Inception to July 11, 2013 | 11 | 0 | 213 | Mostly chronic Stroke | VE  +  GC | Additive to CT  or compared CT or robotic device or no intervention | LL, gait recovery and balance or a mixture | 10/11 studies positive |
| **Lohse et al., 2014**  **(51)** | Inception to April 4, 2013 | 26 | 0 | 626 | 0.04 -6.01 years  (14 chronic) | VE  +  CG  (4) | Additive to CT or robotic device  or compared CT or no intervention | LL motor recovery, UL recovery, gait recovery, balance, cognitive training | VR (VE or CG)  effective across ICF domains (BF, ACT & PART) effect size moderate |
| **Rodrigues-Baroni et al., 2014**  **(47)** | Inception to July 2013 | 7 | 0 | 154 | Chronic stroke | VE  +  CG | CT or none | Gait recovery | VR effective in increasing walking speed |
| **Aguiar Dos Santos et al., 2015**  **(44)** | up to March 31, 2014 | 5 | 0 | 91 | Mostly more than 6 months | CG  (NW) | Additive to CT  Vs CT | UL, motor function, balance, and functionality | VR Improved motor function but not static balance or functional independence |
| **Luque-Moreno et al., 2015**  **(49)** | January 2004 and January 2014 | 11 | 0 | 231 | more than 6 months | 10: VE  1:  CG | Additive to CT or robotic device  or compared to CT | Lower limb motor recovery, gait recovery and balance or a mixture | VR  (>10 sessions) improved gait speed, balance and motor function,  best when combined with CT |
| **Cheok et al., 2015**  **(43)** | Inception to July 2014 | 6 | 0 | 166 | 4= and  2 acute or subacute | CG:  NW only | Additive to CT or compared CT | Gait recovery, balance and functional independence | When additive to CT, NW improves in TUG and not in the other physical measures. Effect size small |
| **Corbetta et al., 2015**  **(25)** | Inception to August 2014, | 15 | 0 | 341 | Majority chronic | VE  +  CG | Additive to CT or compared CT | Walking speed, balance, mobility | When replaces part or all of CT VR Improves walking speed, balance, mobility & TUG test.  When additive, improved TUG |
| **Chen et al., 2016**  **(45)** | January 2006 and December 2015 | 9 | 0 | 265 | Mostly chronic (2 subacute) | VE  + CG | Additive to CT or compared CT or no intervention | Balance recovery | 8/9 significant improvement in static or dynamic balance evidence= moderate |
| **de Rooij et al., 2016**  **(52)** | Inception up to December 1, 2015. | 21 | 0 | 513 | 12.7 days and 11.6 years | VE  + CG | Additive to CT or compared to CT | balance and gait recovery | VR superior to CT.  When additive to CT more benefit particularly in gait and balance when compared to duration and dose matched CT. |
| **Li et al., 2016**  **(46)** | Inception May 2015 | 16 | 0 | 428 | 4 Acute/Subacute and 12 Chronic | VE  +CG | Additive to CT or compared to CT | Balance recovery | Improvement in balance and TUG compared with control |
| **Gibbons et al., 2016**  **(74)** | Inception to  August 2015 | 22 | 0 | 552 | Acute–subacute  Chronic | CE | Majority VR vs CT | Functional balance, static balance, functional gait/mobility, spatiotemporal gait parameters  or motor function | Significant improvement in functional balance, gait velocity, and stride length in the VR group as compared to CT for the chronic group.  No differences between groups in motor function, gait, and functional mobility |
| **dos Santos Palma et al., 2017**  **(44)** | Inception to 16 June 2015 | 54 | 0 | 1811 | 39=chronic, 7=subacute, or 7=acute stroke,1 both acute and subacute | VE  +  CG | CT or no therapy | UL and/or LL and/or balance | +ve on BF and BS. inconclusive on ACT &PART |
| **Iruthayarajah et al., 2017**  **(53)** | 2009 – September 2015 | 17 | 0 | 469 | Chronic stroke | Nintendo® Wii Fit balance board (7), VE | Majority additive to CT vs CT alone | Dynamic and static balance | Compared to CT,  VR was found to improve static and dynamic balance in  chronic stroke patients. Nintendo® Wii Fit balance board may not be effective |
| **Aminov et al., 2018**  **(2)** | inception until 28 June 2017 | 33 | 0 | 971 | 7 =acute-subacute  26=chronic | 19 VE  14 CG | Additive to CT or compared with CT-time matched controls (in 21 /33) | UL and cognition | VR +ve on BF, BS, ACT & cognition (effect size small to medium) but not on PART best results when utilizing purpose-designed VR systems.  Conclusion: evidence supports VR use as an adjunct to CT |
| **Laver et al., 2017**  **(21)** | Inception to April 2017 | 72 | 0 | 2470 | All stages post stroke (majority chronic) | VE  +  CG | Additive to CT or compared CT or no intervention | UL  gait speed, balance,  and ADL | improvement in ADL compared to same dose CT =but not UL, gait speed or balance  When additive (increasing dose of therapy) improvement in UL recovery. |
| **Ahn etl., 2019**  **(75)** | January 1, 2007, to August 31 2017 | 34 | 0 | 1507 | Acute, subacute, and chronic phases post-stroke | 20 VR-based CG, 6 – Wii and Xbox  2 -Robots  Other-Nonimmersive VE | Additive to CT vs CT alone or intervention alone vs CT alone | UL function and independence in ADL | VR intervention is more effective than CT in improving UL function and independence in ADL |
| **Aramaki., 2019**  **(76)** | 2011 to April 2018 | 9 | 4 | 353 | 2 Acute, 11 Chronic | 8 Nintendo Wii, 3 Xbox 360, 1 combination of both, and the other combination of Xbox 360 and Playstation SeeMee | Additive to CT vs CT alone or intervention alone vs CT alone | Balance (6), UL motor function (5), quality of life (4) and ADL (3) | VR is more efficient in the dynamic balance outcome (3 studies). Other studies indicate no difference between outcomes. Overall, there are differences in the results and the evidence is not sufficient to support the use of VR over CT |
| **Mohammadi et al., 2019**  **(77)** | January 2000 till  August 2017 | 14 | 0 | 367 | Acute (2)  Subacute (1)  Chronic (11) | VE, Wii Fit Balance board | VR in combination with CT vs CT alone (majority) | Balance | Significant  improvement was observed in the experimental group compared to control group  with a medium effect size of .64. VR combined with CT is more effective in improving balance than CT alone |
| **De Keersmaecker et al., 2019**  **(78)** | Inception until 4^th^ October 2017 for PubMed and and Web of Science and until 11^th^ January 2018 for Cochrane Central Register of Controlled Trials | 10 | 2 | 219 | Chronic (9 studies)  Acute (2 studies)  Unknown (1) | 2 Fully immersive VE, 1 semi-immersive VE and the rest non-immersive VE | VR + Treadmill vs just treadmill (CT) | Gait function | VR-enhanced gait training is more effective than an identical gait training without VR to improve spatiotemporal gait parameters (i.e. walking speed, cadence, step length, stride length, single limb support period) and functional gait parameters (i.e. Timed Up and Go) in people post-stroke |
| **Ghai et al., 2020**  **(79)** | Inception until August 2019 | 20 | 12 | 809 | Acute, subacute, and chronic phases post-stroke | VE adjunct with treadmill (8)  VR with robot-assisted training (6) | Additive to the CT vs CT alone or VR alone vs CT alone  VR + RAGT vs RAGT only (3) | Gait performance | Significant  enhancements in gait parameters were observed with VR-based interventions compared with conventional therapy |
| **Dominguez-Tellez et al., 2020**  **(80)** | 2007 – March 2018 | 20 | 0 | 874 | Acute, subacute, and chronic phases post-stroke | Immersive and non-immersive VE including CG | Additive to the CT vs CT alone or VR alone vs CT alone | UL motor function and quality of life | VR seems to be effective for the improvement of motor function of UL and quality of life after stroke |
| **Karamians et al., 2020**  **(81)** | January  2005 to May 2019 | 26 | 12 | 1198 | Acute (8)  Chronic (25)  Mixed (3)  Unknown (2) | VE or VE + gaming | VR or Gaming vs CT | UL function | VR or gaming interventions produced an improvement of 28.5% of the maximal possible improvement. A gaming component resulted in a significantly larger treatment gain than just visual feedback. VR or gaming interventions showed a significant treatment  advantage (10.4%) over CT |
| **Mekbib et al., 2020**  **(82)** | 2010 to March 2019 | 27 | 0 | 1094 | Subacute (12)  Chronic (14)  Unknown (1) | VE and CG | Additive to the CT vs CT alone (majority) or VR alone vs CT alone | UL function | Statistically significant improvement in the recovery of UL function in the VR group as compared to the CT group. Patients in the subacute phase of  stroke may benefit from VR therapies more than patients in  chronic phases of stroke |
| **Pintado-Izquierdo et al., 2020**  **(83)** | from January 2005 to June 2020 | 0 | 18 | 479 | Acute/Subacute (4)  Chronic (13)  All phases (1) | CG – SVR, Nintendo Wii, Wii Fit Plus, Wii Balance Board | Additive to CT vs CT alone or CG vs CT alone | Balance and gait | In 10 of 17 studies balance was improved in the interventional group as compared to the CT group. 6 of the 7 studies that studied gait revealed that improvement in gait was greater in the VR group as compared to the experimental group |
| **Amirthalingam et al., 2021**  **(84)** | January 2016 to April 30, 2021 | 13 | 0 | 298 | Acute, subacute, and chronic phases post-stroke | Immersive VE, Nintendo Wii (1) | CT alone, 1 study with additive to CT vs CT alone | UL function, cognitive function, gait, and balance | VR-based rehabilitation more effective than CT in improving UL function, gait, and postural balance post-stroke |
| **Cao et al., 2021**  **(54)** | Inception to September 2020 | 5 | 0 | 47 | Chronic | VE, semi-immersive VE (EVA park), Gaming system for aphasia, ELT within VE | Additive to CT vs CT alone or VE alone vs CT alone | Functional communication | No significant differences between VR and the control groups |
| **Doumas et al., 2021**  **(55)** | Inception till May 5^th^ 2020 | 42 | 0 | 2083 | 1/3 of studies – Subacute  2/3 of studies – chronic | Serious games across different devices (immersive VR, robotic exoskeleton, Microsoft Kinect etc.) | Additive to the CT vs CT alone or VR alone vs CT alone | UL motor function, activity and participation | Rehabilitation through serious games, targeting UL recovery after stroke, leads to better improvements, compared to conventional treatment, in three ICF-WHO components (motor function, activity, and participation) |
| **Cortes-Perez et al., 2021 (56)** | Inception to January 2021 | 2 | 0 | 91 | Unclear | Leap Motion Controller based video games | LMC + CT VS CT alone (1)  LMC vs CT (1) | UL motor function | A higher effect of LMC when combined with CT in improving UL motor function as compared to CT alone |
| **Gao et al., 2021**  **(85)** | Inception to May 31, 2021 | 6 | 0 | 209 | Chronic stroke | Semi- immersive VR (motion tracking, Kinect etc.) | VR + CT vs CT alone | Global and overall cognition, attention, execution, motor function, mood, and ADL | VR-based intervention combined with traditional  rehabilitation showed better outcomes for overall cognition, attention/execution, and  depressive mood in individuals with chronic stroke. Non-significant effect for global  cognition, motor function, and ADL in individuals with chronic stroke |
| **Garay-Sachez et al., 2021 (57)** | December 2010 to December 2020 | 10 | 0 | 316 | Acute and subacute | Immersive and non-immersive VR | VR alone vs CT alone (1)  VR + CR vs CT alone | Dynamic and static balance | Static balance: 4 studies showed significant improvement for non-immersive VR over CT and 1 for immersive VR  Dynamic balance: 4 studies using non-immersive VR in combination with CT showed significant improvement and 2 studies using immersive VR in combination with CT showed significant improvement |
| **Khan et al., 2021 (61)** | 2011–2020 | 18 | 55 | 1617 | All phases post stroke | VE, rehabilitation gaming system, Nintendo Wii | VR + CT vs CT alone or VR vs CT or VR vs no therapy | Motor, sensory, and cognitive outcomes | Improved functional outcomes reported by studies in all 3 outcomes measured, but meta-analysis done revealed no statistically significant difference  compared to CT. |
| **Palacios-Navarro et al., 2021 (86)** | Inception until 31^st^ October 2020 | 8 | 0 | 1472 | Chronic phase | Fully immersive VE | Immersive VE + CT vs CT alone/Non-immersive VE | UL motor function, gait, balance | UL Function: Significant improvement in the intervention group compared to the control, exhibiting a large effect size (0.79) VR intervention training achieved significantly faster walking speed compared to CT. Overall significant improvements in favor of the immersive VR group |
| **Peng et al., 2021**  **(62)** | Inception  until October 10, 2020, | 17 | 2 | 681 | Subacute Stroke | VE, CG based VR, | Additive to CT vs CT or VR/CG vs CT alone | Motor function (UL + LL) | When compared to CT, VR resulted in mild improvement in motor function (SMD = 0.47;  95% CI = 0.22–0.72; I  2 = 75%; P < 0.001). Upon trim-and-fill adjustment, this  finding was deemed insignificant |
| **Zhang et al., 2021 (87)** | Inception until 31^st^ December 2019 | 87 | 0 | 3540 | All phases were included | VE | Additive to CT vs CT alone or VR vs CT alone | UL and LL motor function, balance, gait, cognition, and daily function | “VR improves limb function, walking ability, balance, gait velocity, cadence,  and daily life activities to a greater extent than CT. However, VR had a similar effect on improvement of cognition as CT therapy” |
| **Zhang et al., 2021 (58)** | Inception to April 15, 2021 | 23 | 0 | 894 | Unspecified | Immersive, semi-immersive and non-immersive VE, CG | VR alone vs CT alone (16), VR + CT vs CT (5), VR + Computer based cognition vs CT (2) | Global cognition and  domain-specific cognition (attention, executive function,  memory, psychomotor speed, verbal fluency) | VR-based therapies are more efficacious in improving executive function,  memory, and visuospatial function post stroke than CT. No significant differences were found between the 2 groups in terms of global cognitive function, attention, verbal fluency, depression,  and the quality of life |
| **Aguilera-Rubio et al., 2022 (88)** | 2012 to December,2020 | 2 | 4 | 144 | 2 studies – Subacute phase of stroke  1 study – Acute phase  1 study – Subacute and acute phase  1 study – chronic  1 study - unspecified | Leap Motion Controller | Additive to CT or compared CT or no intervention | UL | Statistically significant improvement in UL functionality (5 studies), grip strength (4 studies), spasticity (1), dexterity, performance, participation, satisfaction, and usability. Further research needed due to heterogeneity |
| **Al-Whaibi et al., 2022 (89)** | 1 search from inception to June 25, 2020  Another search done on 1^st^ Feb, 2021 | 6 | 0 | 174 | Chronic stroke | VE | CT alone | UL Motor function | No statistical difference in UL performance in VR group as compared to the CT group |
| **Chan et al., 2022 (63)** | Inception until January 2021 | 32 | 2 | 900 | Chronic stroke | Exergaming, type of VR | Additive to CT vs CT alone (30)  No intervention (2) | Balance, LL  functional mobility and functional independence | Exergaming shows statistically significant improvement in balance, lower limb  functional mobility and functional independence among individuals with chronic  stroke as compared to CT alone |
| **Chen et al., 2022**  **(60)** | Inception to December 31, 2021 | 42 | 0 | 1893 | Subacute and Chronic stroke | Specialized VE and CG | VR alone vs CT alone OR VR alone vs No intervention OR VR + CT vs CT alone | UL Motor Rehabilitation | Statistically significant improvement in UL motor function, muscle strength, range of motion and independence in day-to-day activities |
| **Chen et al., 2022**  **(59)** | Inception until August 31, 2021 | 21 | 0 | 1149 | Unclear | VE | Additive to the CT vs CT alone or VR alone vs CT alone | Cognitive function and ADL | VR training improved cognitive function and ADL in PSCI compared to CT |
| **Fernandez-Vazquez et al., 2022**  **(50)** | Inception to May 2022 | 7 | 0 | 230 | Acute phase (3) Chronic phase (4) | Haptic Glove Systems in Combination with Semi-Immersive VR | Additive to the CT vs CT alone or VR alone vs CT alone | UL Motor Rehabilitation | Combination of rehabilitation haptic gloves, SVR, and CT produces significant improvement in UL functionality as compared to CT alone |
| **Hao, J., Buster, T., 2022**  **(90)** | Inception to September 8^th^, 2022 | 8 | 0 | 190 | Chronic | VE | VR + treadmill training vs treadmill training only | Walking speed and endurance, balance function, number of falls | Virtual reality augmented treadmill walking training enhances outcomes compared to treadmill-only training in patients with walking and balance impairments |
| **Hao et al., 2022**  **(91)** | 2000 to October 17, 2021 | 17 | 0 | 921 | Acute stroke | VE (Nintendo Wii, Microsoft Kinect) | Additive to the CT vs CT alone or VR alone vs CT alone | UL function, cognitive function (5), gait speed, ADL, and balance ability | Effects of VR are  comparable to conventional rehabilitation, no differences  between VR and dose-matched conventional rehabilitation on UL function, ADL  outcomes, balance function, and cognition |
| **Leong et al., 2022**  **(92)** | Inception to October 15, 2021 | 50 | 0 | 2271 | Acute (4)  Subacute (9)  Chronic (26) | Virtual, augmented, and mixed reality (VAMR) | Additive to the CT vs CT alone or intervention alone vs CT alone | UL function and ADL | VAMR therapy was superior to CT in UL impairment and daily function outcomes, but not UL function measures. Patients with chronic stroke significantly improved better than those with subacute after VAMR training |
| **Li et al., 2022**  **(73)** | Inception to  May 24, 2021 | 31 | 0 | 1299 | Acute (1)  Subacute (8)  Chronic (21) | VE, CG | VR + CT vs Time-dosed matched CT or VR only vs Time-dosed matched CT only or CG only vs time-dosed matched CT | ICF domains: body structure or function, activity, and participation: UL function, | VR is more superior to time-dose matched CT in terms of recovery of upper extremity motor function in patients poststroke, especially when VE is used, or VR is combined with CT. VR does not improve patients’ daily activity performance  and participation compared with CT |
| **Mugisha et al., 2022**  **(93)** | 2015 to May 2020 | 22 | 0 | 1253 | Unspecified | Immersive and Non-immersive VE using Nintendo Wii, Miscrosoft Kinect etc. | Additive to the CT vs CT alone or intervention alone vs CT alone | UL activity and function, LL activity and function, balance, activity of daily life, adverse events | No statistical difference between VR and CT in improving UL and LL motor function, balance, and ADL. Immersive VE is superior to non-immersive VE in improving the outcomes measured |
| **Parisi et al., 2022**  **(35)** | Inception to 17 January 2022 | 10 | 0 | 283 | Acute (2)  Subacute (4)  Chronic (3) | VE/VE + Motion tracking | Additive to the CT vs CT alone or intervention alone vs CT alone | Cognitive rehabilitation | VR without motion tracking was more effective than CT |
| **Sevcenko & Lindgren, 2022**  **(94)** | Inception to February 29, 2020 | 10 | 0 | 715 | Subacute (4)  Chronic (4)  Both of the above (2) | VE | Additive to the CT vs CT alone or intervention alone vs CT alone | Functional ability (UL, gait, balance) | VR training is suggested as an effective intervention to improve the functional ability in stroke especially when combined with CT. Some studies showed significant improvement of VR group in gait, balance, quality of life and fatigue while no effect was seen in the CT group. |
| **Wang et al., 2022**  **(95)** | Inception to December 2021 | 24 | 0 | 793 | Acute (6)  Subacute (5)  Chronic (13) | Game-based non-immersive VR, CG and Custom games were included | Additive to the CT vs CT alone or intervention alone vs CT alone | UL rehabilitation, hand dexterity,  daily living ability, and cognitive function | Game-based VR UL rehabilitation therapy for cerebral apoplexy is more effective than CT in improving  patients’ UL function and hand mobility |
| **Wiley et al., 2022**  **(34)** | Inception to November 13th 2019 | 8 | 0 | 196 | Majority in the chronic phased | Immersive, non-immersive and semi-immersive VE | Majority combination of VE + CT vs CT alone | Cognition, executive function, language, and memory | VR therapy was not more effective than control for  improving global cognition and attention |
