# Supplementary material for: Virtual reality in stroke recovery: a meta-review of systematic reviews
Source: Bioelectron Med. 2024 Oct 5;10:23. doi: 10.1186/s42234-024-00150-9 (PMC11452980; doi:10.1186/s42234-024-00150-9)
Supplement: Supplementary file 2 — Supplementary Material 2: Table S2. Quality ratings of the systematic reviews using the AMSTAR-2 tool. [file 42234_2024_150_MOESM2_ESM.docx]

Table S2. Quality rating of the systematic reviews using the AMSTAR 2 tool

| **Author** | **AMSTAR 2 rating** | **Critical**  **domain**  **missing** | **Comment** |
| --- | --- | --- | --- |
| **Crosbie et al., 2007**  **(39)** | Critically Low | 2,7,9,13 | Multiple critical domains |
| **Henderson et al., 2007**  **(40)** | Low | 7 | 4,2= Partial yes and one non-critical item |
| **Saposnik and Levin, 2011**  **(32)** | Critically Low | 7 ,9 | 3, 4 =partial yes and multiple (4) non -critical items |
| **Smith et al., 2012**  **(41)** | Moderate |  | 7,2 =partial yes and multiple (2) non -critical items (downgraded to moderate) |
| **Cavalcanti Moreira et al., 2013**  **(42)** | Low | 7 | 2,4 =partial yes |
| **Casserly and Baer, 2014**  **(19)** | Low |  | 2=Partial yes and multiple (4) non -critical items, (downgraded to low) |
| **Thomson et al., 2014**  **(18)** | Low | 7 | 2=Partial yes and multiple (3) non- critical items |
| **Imam and Jarus, 2014 (40)** | Low | 13 | 4,2= Partial yes |
| **Lohse et al., 2014**  **(51)** | High |  | one non -critical items (item 16 not mentioned in the published paper, but was retrieved from PROSPERO registration) |
| **Rodrigues-Baroni et al., 2014**  **(47)** | Critically Low | 7,15 | 2=Partial yes and multiple (2) non- critical items |
| **Aguiar Dos Santos et al., 2015**  **(44)** | Critically Low | 2,7 | 4=Partial yes and multiple (3) non- critical items |
| **Luque-Moreno et al., 2015**  **(49)** | Low | 7 | 2=Partial yes and multiple (4) non- critical items, |
| **Cheok et al., 2015**  **(43)** | Low | 15 | 2=Partial yes and multiple (2) non- critical items |
| **Corbetta et al., 2015**  **(25)** | Low | 7 | 2 =partial yes and one non-critical item |
| **Chen et al., 2016**  **(45)** | Low | 7 | 4,2= Partial yes and multiple (2) non- critical items |
| **de Rooij et al., 2016**  **(52)** | Moderate |  | 2 =partial yes and multiple (2) non -critical items (downgraded to moderate) |
| **Li et al., 2016**  **(46)** | Low | 15 | 4= Partial yes and one non-critical item |
| **Gibbons et al., 2016**  (74) | Low | 2 | Multiple (6) non-critical domains |
| **dos Santos Palma et al., 2017**  **(44)** | Low | 7 | 8,4,2= Partial yes and multiple (2) non- critical items |
| **Iruthayarajah et al., 2017**  **(53)** | Critically Low | 2, 13, 15 | 7, 11, 14 = partial yes and multiple (2) non-critical domains |
| **Aminov et al., 2018**  **(2)** | High |  | 2,7 =partial yes and one non-critical item |
| **Laver et al., 2017**  **(21)** | High |  | one non -critical items |
| **Ahn etl., 2019**  **(75)** | Low | 2 | 7, 9 = Partial yes and one non-critical item |
| **Aramaki., 2019**  **(76)** | Critically Low | 2, 9, 13 | 1, 4, 7 = Partial yes and multiple (2) non-critical items |
| **Mohammadi et al., 2019**  **(77)** | Critically Low | 2, 15 | 4, 7 = Partial yes and multiple (2) non -critical items |
| **De Keersmaecker et al., 2019**  **(78)** | Low | 15 | 5, 12 = Partial yes and (2) non -critical items |
| **Ghai et al., 2020**  **(79)** | Low | 2 | 3, 4, 7 = Partial yes |
| **Dominguez-Tellez et al., 2020**  **(80)** | Critically Low | 2, 15 | 3, 4, 7, 12 = Partial yes and multiple (3) non-critical items |
| **Karamians et al., 2020**  **(81)** | Low | 15 | 4, 7 = Partial yes and multiple (2) non -critical items |
| **Mekbib et al., 2020**  **(82)** | Critically Low | 2, 15 | 2, 4, 7, 8 = Partial yes and multiple (2) non -critical items |
| **Pintado-Izquierdo et al., 2020**  **(83)** | Critically Low | 2, 15 | 7 = Partial yes and multiple (2) non -critical items |
| **Amirthalingam et al., 2021**  **(84)** | Low | 2 | 3, 4, 7 = Partial yes and multiple (3) non -critical items |
| **Cao et al., 2021**  **(54)** | Moderate |  | 2, 4, 7, 15 = Partial yes |
| **Doumas et al., 2021**  **(55)** | High |  | 3, 7 = Partial yes and (1) non -critical item |
| **Cortes-Perez et al., 2021 (56)** | High |  | 3, 7 = Partial yes and (1) non -critical item |
| **Gao et al., 2021**  **(85)** | Critically Low | 2, 15 | 7, 12, 14 = Partial yes and (1) non-critical items |
| **Garay-Sachez et al., 2021 (57)** | Moderate |  | 3, 4, 7, 13 = partial yes and multiple (2) non -critical items (downgraded to moderate) |
| **Khan et al., 2021 (61)** | Critically Low | 2, 15 | 3 = Partial yes and multiple (2) non -critical items |
| **Palacios-Navarro et al., 2021 (86)** | Low | 2 | 7, 13 = Partial yes and multiple (2) non-critical item |
| **Peng et al., 2021**  **(62)** | Low | 2 | 3, 7 = Partial yes and one non-critical item |
| **Zhang et al., 2021 (87)** | Low | 15 | 3, 4, 7 = Partial yes and one non-critical item |
| **Zhang et al., 2021 (58)** | High |  | 3, 7 =partial yes and one non-critical item |
| **Aguilera-Rubio et al., 2022 (88)** | Low | 2 | 4, 7, 14 = Partial yes and one non-critical item |
| **Al-Whaibi et al., 2022 (89)** | Low | 2 | One non-critical item |
| **Chan et al., 2022 (63)** | Low | 2 | 3, 13, 14 = Partial yes and one non-critical item |
| **Chen et al., 2022**  **(60)** | High |  | 3 = Partial yes and (1) non -critical item |
| **Chen et al., 2022**  **(59)** | Low | 2 | 4, 7 = Partial yes and one non-critical item |
| **Fernandez-Vazquez et al., 2022**  **(50)** | High |  | 3, 4, 13 = partial yes and one non-critical item |
| **Hao, J., Buster, T., 2022**  **(90)** | Low | 15 | 3, 4, 12, 13, 14 = partial yes and one non-critical item |
| **Hao et al., 2022**  **(91)** | Low | 15 | 3, 4, 13, 14 = Partial yes and one non -critical items |
| **Leong et al., 2022**  **(92)** | Critically Low | 2, 13 | 3, 7, 12, 14 =partial yes and one non -critical item |
| **Li et al., 2022**  **(73)** | Low | 2 | 3, 7 = Partial yes and one non -critical items |
| **Mugisha et al., 2022**  **(93)** | Low | 15 | 3 = Partial yes and one non -critical items |
| **Parisi et al., 2022**  **(35)** | High |  | 4, 14 = partial yes and one non-critical item |
| **Sevcenko & Lindgren, 2022**  **(94)** | Low | 2 | 3, 5, 6, 7 = Partial yes and one non -critical item |
| **Wang et al., 2022**  **(95)** | Critically Low | 2, 15 | 3, 4, 7, 14 =Partial yes and one non- critical item |
| **Wiley et al., 2022**  **(34)** | Critically Low | 2, 15 | 3, 14 =Partial yes and one non- critical item |
