# Supplementary material for: Virtual reality in stroke recovery: a meta-review of systematic reviews
Source: Bioelectron Med. 2024 Oct 5;10:23. doi: 10.1186/s42234-024-00150-9 (PMC11452980; doi:10.1186/s42234-024-00150-9)
Supplement: Supplementary file 3 — Supplementary Material 3. [file 42234_2024_150_MOESM3_ESM.docx]

**Table 1: Appendix 1. Search strategies:**

**1.Medline (PubMed): 12/05/2022**

|  | **Syntax** | **Hits** |
| --- | --- | --- |
| #1 | Search ((stroke[Title/Abstract] OR cerebral vascular Accident[Title/Abstract] OR hemiplegia[Title/Abstract])) AND (cognition[Title/Abstract] OR cognitive impairment[Title/Abstract] OR memory[Title/Abstract] OR memory impairment[Title/Abstract] OR dementia[Title/Abstract] OR attention[Title/Abstract] OR attention deficit [Title/Abstract] OR neglect[Title/Abstract] OR executive function[Title/Abstract] OR language[Title/Abstract] OR aphasia[Title/Abstract] OR motor function [Title/Abstract] OR muscle weakness[Title/Abstract] OR upper limb[Title/Abstract] OR lower limb[Title/Abstract] OR gait[Title/Abstract] OR walking[Title/Abstract] OR balance[Title/Abstract]) | 45,718 |
| #2 | Search (Virtual reality [Title/Abstract] OR virtual [Title/Abstract] OR game based virtual reality[Title/Abstract] OR virtual environment,[Title/Abstract] OR augmented reality[Title/Abstract] OR computer based virtual reality[Title/Abstract]) | 93,828 |
| #3 | Search (((Virtual reality[Title/Abstract] OR virtual[Title/Abstract] OR game based virtual reality[Title/Abstract] OR virtual environment,[Title/Abstract] OR augmented reality[Title/Abstract] OR computer based virtual reality[Title/Abstract]))) AND (((stroke[Title/Abstract] OR cerebral vascular Accident[Title/Abstract] OR hemiplegia[Title/Abstract])) AND (cognition[Title/Abstract] OR cognitive impairment[Title/Abstract] OR memory[Title/Abstract] OR memory impairment[Title/Abstract] OR dementia[Title/Abstract] OR attention[Title/Abstract] OR attention deficit [Title/Abstract] OR neglect[Title/Abstract] OR executive function[Title/Abstract] OR language[Title/Abstract] OR aphasia[Title/Abstract] OR motor function [Title/Abstract] OR muscle weakness[Title/Abstract] OR upper limb[Title/Abstract] OR lower limb[Title/Abstract] OR gait[Title/Abstract] OR walking[Title/Abstract] OR balance[Title/Abstract])) | 912 |
| #4 | Search (((Virtual reality[Title/Abstract] OR virtual[Title/Abstract] OR game based virtual reality[Title/Abstract] OR virtual environment,[Title/Abstract] OR augmented reality[Title/Abstract] OR computer based virtual reality[Title/Abstract]))) AND (((stroke[Title/Abstract] OR cerebral vascular Accident[Title/Abstract] OR hemiplegia[Title/Abstract])) AND (cognition[Title/Abstract] OR cognitive impairment[Title/Abstract] OR memory[Title/Abstract] OR memory impairment[Title/Abstract] OR dementia[Title/Abstract] OR attention[Title/Abstract] OR attention deficit [Title/Abstract] OR neglect[Title/Abstract] OR executive function[Title/Abstract] OR language[Title/Abstract] OR aphasia[Title/Abstract] OR motor function [Title/Abstract] OR muscle weakness[Title/Abstract] OR upper limb[Title/Abstract] OR lower limb[Title/Abstract] OR gait[Title/Abstract] OR walking[Title/Abstract] OR balance[Title/Abstract])) Filters: Review | 148 |
| #5 | Search (((Virtual reality[Title/Abstract] OR virtual[Title/Abstract] OR game based virtual reality[Title/Abstract] OR virtual environment,[Title/Abstract] OR augmented reality[Title/Abstract] OR computer based virtual reality[Title/Abstract]))) AND (((stroke[Title/Abstract] OR cerebral vascular Accident[Title/Abstract] OR hemiplegia[Title/Abstract])) AND (cognition[Title/Abstract] OR cognitive impairment[Title/Abstract] OR memory[Title/Abstract] OR memory impairment[Title/Abstract] OR dementia[Title/Abstract] OR attention[Title/Abstract] OR attention deficit [Title/Abstract] OR neglect[Title/Abstract] OR executive function[Title/Abstract] OR language[Title/Abstract] OR aphasia[Title/Abstract] OR motor function [Title/Abstract] OR muscle weakness[Title/Abstract] OR upper limb[Title/Abstract] OR lower limb[Title/Abstract] OR gait[Title/Abstract] OR walking[Title/Abstract] OR balance[Title/Abstract])) Filters: Review; Humans | 148 |
| #6 | Search (((Virtual reality[Title/Abstract] OR virtual[Title/Abstract] OR game based virtual reality[Title/Abstract] OR virtual environment,[Title/Abstract] OR augmented reality[Title/Abstract] OR computer based virtual reality[Title/Abstract]))) AND (((stroke[Title/Abstract] OR cerebral vascular Accident[Title/Abstract] OR hemiplegia[Title/Abstract])) AND (cognition[Title/Abstract] OR cognitive impairment[Title/Abstract] OR memory[Title/Abstract] OR memory impairment[Title/Abstract] OR dementia[Title/Abstract] OR attention[Title/Abstract] OR attention deficit [Title/Abstract] OR neglect[Title/Abstract] OR executive function[Title/Abstract] OR language[Title/Abstract] OR aphasia[Title/Abstract] OR motor function [Title/Abstract] OR muscle weakness[Title/Abstract] OR upper limb[Title/Abstract] OR lower limb[Title/Abstract] OR gait[Title/Abstract] OR walking[Title/Abstract] OR balance[Title/Abstract])) Filters: Systematic Review; Meta-Analysis; Humans; English | 73 |
|  | **Results = 73** | |

**2. PEDro 12/05/2022**

Simple search: Search terms: Virtual Reality AND Stroke; limits: Systematic reviews

**Results = 60**

**3. Cochrane Database of Systematic Reviews 12/05/2022**

**ID** Search

#1 MeSH descriptor: [Virtual Reality Exposure Therapy] explode all trees

#2 MeSH descriptor: [Virtual Reality] explode all trees

#3 #1 or #2

#4 MeSH descriptor: [Stroke Rehabilitation] explode all trees

#5 MeSH descriptor: [Stroke] explode all trees

#6 #4 or #5

#7 #3 and #6

#8 "virtual reality":ti,ab,kw or "virtual reality therapy" or "video game" and "stroke" (Word variations have been searched)

#9 #7 or #8 in Cochrane Reviews (Reviews and Protocols)

**Results = 38**


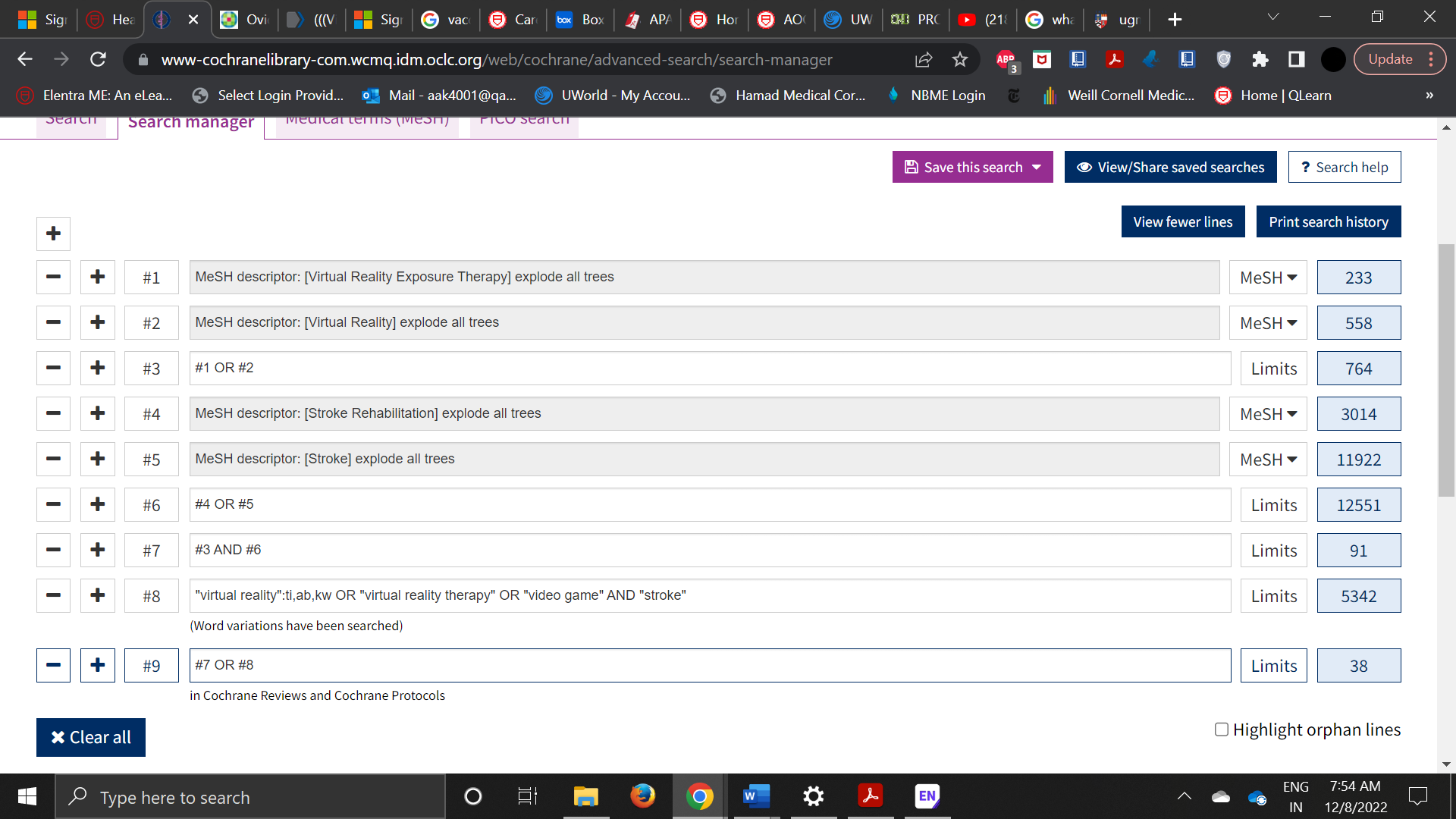


**4. DARE(Database of Abstracts of Reviews of Effects): 12/05/2022**

((stroke OR cerebral vascular Accident OR hemiplegia AND cognition OR cognitive impairment OR memory OR memory impairment OR dementia OR attention OR attention deficit OR neglect OR executive function OR language OR aphasia OR dysphasia OR motor function OR muscle weakness OR upper limb OR lower limb OR gait OR walking OR balance ) AND (Virtual reality OR virtual OR game based virtual reality OR virtual environment OR augmented reality OR computer based virtual reality)) and ((Systematic review:ZDT and Bibliographic:ZPS) OR (Systematic review:ZDT and Abstract:ZPS) OR (Cochrane review:ZDT) OR (Cochrane related review record:ZDT)) IN DARE

**Results= 9**

**5. EMBASE(Ovid):) 12/05/2022**

1 virtual reality/ (25333)

2 (Virtual reality or virtual or game based virtual reality or video gaming or virtual environment or augmented reality or computer based virtual reality).mp. [mp=title, abstract, heading word, drug trade name, original title, device manufacturer, drug manufacturer, device trade name, keyword, floating subheading word, candidate term word] (129860)

3 1 or 2 (129860)

4 exp cerebrovascular accident/ (301814)

5 (stroke or cerebral vascular Accident or hemiplegia).mp. [mp=title, abstract, heading word, drug trade name, original title, device manufacturer, drug manufacturer, device trade name, keyword, floating subheading word, candidate term word] (571552)

6 4 or 5 (643977)

7 exp stroke rehabilitation/ or exp rehabilitation/ or exp rehabilitation research/ or exp cognitive rehabilitation/ or exp speech rehabilitation/ or exp "speech and language rehabilitation"/ or exp rehabilitation medicine/ or exp rehabilitation care/ (484721)

8 (rehabilitation or recovery).mp. [mp=title, abstract, heading word, drug trade name, original title, device manufacturer, drug manufacturer, device trade name, keyword, floating subheading word, candidate term word] (1131767)

9 7 or 8 (1402865)

10 exp cognition assessment/ or exp cognition/ (2938648)

11 (cognition or cognitive or memory).mp. [mp=title, abstract, heading word, drug trade name, original title, device manufacturer, drug manufacturer, device trade name, keyword, floating subheading word, candidate term word] (1154509)

12 10 or 11 (3382867)

13 exp attention/ or exp visual attention/ or exp attention disturbance/ (343218)

14 (attention or neglect).mp. [mp=title, abstract, heading word, drug trade name, original title, device manufacturer, drug manufacturer, device trade name, keyword, floating subheading word, candidate term word] (768459)

15 13 or 14 (939082)

16 exp frontal lobe/ or exp executive function/ or exp task performance/ (394408)

17 (executive function or task performance).mp. [mp=title, abstract, heading word, drug trade name, original title, device manufacturer, drug manufacturer, device trade name, keyword, floating subheading word, candidate term word] (217969)

18 16 or 17 (406521)

19 exp aphasia/ (34684)

20 (language or aphasia or dysphasia).mp. [mp=title, abstract, heading word, drug trade name, original title, device manufacturer, drug manufacturer, device trade name, keyword, floating subheading word, candidate term word] (337753)

21 19 or 20 (339268)

22 exp motor performance/ (89811)

23 (motor function or upper limb power or upper limb function or lower limb power or lower limb function or muscle power).mp. [mp=title, abstract, heading word, drug trade name, original title, device manufacturer, drug manufacturer, device trade name, keyword, floating subheading word, candidate term word] (52701)

24 22 or 23 (115961)

25 exp gait/ (67478)

26 (gait or walking or Balance).mp. [mp=title, abstract, heading word, drug trade name, original title, device manufacturer, drug manufacturer, device trade name, keyword, floating subheading word, candidate term word] (622575)

27 25 or 26 (622575)

28 12 or 15 or 18 or 21 or 24 or 27 (4687563)

29 3 and 6 and 28 (2051)

30 29 and "Review".sa_pubt. (273)

**Results = 273**

|  | **Syntax** | **Hits** |
| --- | --- | --- |
| 1 | exp Virtual Reality/ | 7143 |
| 2 | Virtual reality.mp. [mp=title, abstract, heading word, table of contents, key concepts, original title, tests & measures] | 8465 |
| 3 | exp Cerebrovascular Accidents/ or exp Cerebral Ischemia/ | 22070 |
| 4 | (Stroke or cerebrovascular accident).mp. [mp=title, abstract, heading word, table of contents, key concepts, original title, tests & measures] | 31363 |
| 5 | 1 or 2 | 8465 |
| 6 | 3 or 4 | 34531 |
| 7 | 5 and 6 | 232 |
| 8 | limit 7 to (human and English language and human) | 226 |
| 9 | limit 8 to "0800 literature review" | 27 |
| 10 | limit 9 to "0830 systematic review" | 13 |
| 11 | limit 10 to 1200 meta-analysis | 2 |
| 12 | 9 or 10 or 11 | 42 |
|  | **Results = 42** | |

**6. PsycINFO (via Ovid) last updated 12/05/2022**

TX virtual reality OR TI Virtual reality AND (cerebrovascular accident OR cerebral ischemia OR TX stroke)

Limiters: English, Systematic Review, Meta-analysis

**7.Web of Science last updated 12/05/2022**

|  | **Syntax** | **Hits** |
| --- | --- | --- |
| #1 | **TOPIC:** (virtual reality)  *DocType=All document types; Language=All languages* | 71243 |
| #2 | **TITLE:** (Virtual reality)8.  *DocType=All document types; Language=All languages;* | 23979 |
| #3 | **TITLE:** (Stroke OR cerebrovascular accident)  *DocType=All document types; Language=All languages;* | 183301 |
| #4 | **TOPIC:** (Stroke or cereberovascular accident)  *DocType=All document types; Language=All languages;* | 432630 |
| #5 | **TOPIC:** (Augmented reality)  *DocType=All document types; Language=All languages;* | 30501 |
| #6 | **TITLE:** (Augmented reality)  *DocType=All document types; Language=All languages;* | 12778 |
| #7 | #6 OR #5 OR #2 OR #1  *DocType=All document types; Language=All languages;* | 8807 |
| #8 | #4 OR #3  *DocType=All document types; Language=All languages;* | 433512 |
| #9 | #8 AND #7  *DocType=All document types; Language=All languages;* | 2805 |
| #10 | #8 AND #7 Refined by: **DOCUMENT TYPES:** (REVIEW)  *DocType=All document types; Language=All languages;* | 420 |
| #11 | #8 AND #7 Refined by: **DOCUMENT TYPES:** (REVIEW) AND **LANGUAGES:** (ENGLISH)  *DocType=All document types; Language=All languages;* | 376 |
|  | **Results = 376** | |

**8.Manual bibliography of included full article search: yielded 0 articles**

Currently,

Total = 863

Duplicates = 242

Title/Abstract Screening = 529

Full text screening = 36

Included = 56
