# Supplementary material for: Virtual reality in stroke recovery: a meta-review of systematic reviews
Source: Bioelectron Med. 2024 Oct 5;10:23. doi: 10.1186/s42234-024-00150-9 (PMC11452980; doi:10.1186/s42234-024-00150-9)
Supplement: Supplementary file 4 — Supplementary Material 4. [file 42234_2024_150_MOESM4_ESM.docx]

**Appendix 2**

**Table S2: AMSTAR 2: A critical appraisal tool for systematic reviews that include randomized or non-randomized studies of healthcare interventions, or both**

| **1.** | **Did the research questions and inclusion criteria for the review include the components of PICO?** | | | |
| --- | --- | --- | --- | --- |
|  |  |  |  |  |
| For Yes: |  | Optional (recommended) |  |  |
|  | Population |  Timeframe for follow-up |  | Yes |
|  | Intervention |  |  | No |
|  | Comparator group |  |  |  |
|  | Outcome |  |  |  |

**2.** **Did the report of the review contain an explicit statement that the review methods were established prior to the conduct of the review and did the report justify any significant deviations from the protocol?**

| For Partial Yes: | | For Yes: |  |  |  |  |  |  |
| --- | --- | --- | --- | --- | --- | --- | --- | --- |
| The authors state that they had a written | | As for partial yes, plus the protocol | | | |  |  |  |
| protocol or guide that included ALL the | | should be registered and should also | | | |  |  |  |
| following: | | have specified: | | | |  |  |  |
|  |  |  |  |  |  |  | Yes |  |
|  | review question(s) |  | a meta-analysis/synthesis plan, | | |  | Partial Yes |  |
|  | a search strategy |  | if appropriate, *and* | | |  | No |  |
|  | inclusion/exclusion criteria |  a plan for investigating causes | | | |  |  |  |
|  | a risk of bias assessment |  | of heterogeneity | | |  |  |  |
|  |  |  justification for any deviations | | | |  |  |  |
|  |  |  |  |  |  |  |  |  |
|  |  |  | from the protocol | | |  |  |  |
| **3.** | **Did the review authors explain their selection of the study designs for inclusion in the review?** | | | | | | |  |
|  | | | |  |  |  |  |  |
| For Yes, the review should satisfy ONE of the following: | | | | | |  |  |  |
|  | *Explanation for* including only RCTs | |  |  |  |  | Yes |  |
|  | OR *Explanation for* including only NRSI | |  |  |  |  | No |  |
|  | OR *Explanation for* including both RCTs and NRSI | | | | |  |  |  |
| **4.** | **Did the review authors use a comprehensive literature search strategy?** | | | | |  |  |  |
|  | |  | |  |  |  |  |  |
| For Partial Yes (all the following): | | For Yes, should also have (all the | | | |  |  |  |
|  |  | following): | | | |  |  |  |
|  | searched at least 2 databases |  | searched the reference lists / | | |  | Yes |  |
|  | (relevant to research question) |  | bibliographies of included | | |  | Partial Yes |  |
|  | provided key word and/or |  | studies | | |  | No |  |
|  | search strategy |  | searched trial/study registries | | |  |  |  |
|  | justified publication restrictions |  | included/consulted content | | |  |  |  |
|  | (e.g. language) |  | experts in the field | | |  |  |  |
|  |  |  where relevant, searched for | | | |  |  |  |
|  |  |  | grey literature | | |  |  |  |
|  |  |  conducted search within 24 | | | |  |  |  |
|  |  |  | months of completion of the | | |  |  |  |
|  |  |  | review | | |  |  |  |
| **5.** | **Did the review authors perform study selection in duplicate?** | | | | |  |  |  |
|  | |  |  |  |  |  |  |  |
| For Yes, either ONE of the following: | |  |  |  |  |  |  |  |
|  | at least two reviewers independently agreed on selection of eligible studies | | | | |  | Yes |  |
|  | and achieved consensus on which studies to include | | | | |  | No |  |
|  | OR two reviewers selected a sample of eligible studies and achieved good | | | | |  |  |  |
|  |  |  |  |  |  |  |  |  |

agreement (at least 80 percent), with the remainder selected by one reviewer.

1. **Did the review authors perform data extraction in duplicate?**

| For Yes, either ONE of the following: | |  |  |  |  |  |
| --- | --- | --- | --- | --- | --- | --- |
|  | at least two reviewers achieved consensus on which data to extract from | | |  | Yes |  |
|  | included studies |  |  |  | No |  |
|  | OR two reviewers extracted data from a sample of eligible studies and | | |  |  |  |
|  | achieved good agreement (at least 80 percent), with the remainder | | |  |  |  |
|  | extracted by one reviewer. |  |  |  |  |  |
| **7.** | **Did the review authors provide a list of excluded studies and justify the exclusions?** | | | | |  |
|  | |  | |  |  |  |
| For Partial Yes: | | For Yes, must also have: | |  |  |  |
|  | provided a list of all potentially |  Justified the exclusion from | |  | Yes |  |
|  | relevant studies that were read |  | the review of each potentially |  | Partial Yes |  |
|  | in full-text form but excluded |  | relevant study |  | No |  |
|  | from the review |  |  |  |  |  |
| **8.** | **Did the review authors describe the included studies in adequate detail?** | | |  |  |  |
|  | |  | |  |  |  |
| For Partial Yes (ALL the following): | | For Yes, should also have ALL the | |  |  |  |
|  |  | following: | |  |  |  |
|  | described populations |  described population in detail | |  | Yes |  |
|  | described interventions |  | described intervention in |  | Partial Yes |  |
|  | described comparators |  | detail (including doses where |  | No |  |
|  | described outcomes |  | relevant) |  |  |  |
|  |  |  described comparator in detail | |  |  |  |
|  | described research designs |  |  |  |  |  |
|  |  |  | (including doses where |  |  |  |
|  |  |  |  |  |  |  |
|  |  |  | relevant) |  |  |  |
|  |  |  | described study’s setting |  |  |  |
|  |  |  timeframe for follow-up | |  |  |  |

1. **Did the review authors use a satisfactory technique for assessing the risk of bias (RoB) in individual studies that were included in the review?**

| **RCTs** |  |  |  |  |  |  |
| --- | --- | --- | --- | --- | --- | --- |
| For Partial Yes, must have assessed RoB | | For Yes, must also have assessed RoB | |  |  |  |
| from |  | from: |  |  |  |  |
|  | unconcealed allocation, *and* |  | allocation sequence that was |  | Yes |  |
|  lack of blinding of patients and | |  | not truly random, *and* |  | Partial Yes |  |
|  | assessors when assessing |  | selection of the reported result |  | No |  |
|  | outcomes (unnecessary for |  | from among multiple |  | Includes only |  |
|  | objective outcomes such as all- |  | measurements or analyses of a |  | NRSI |  |
|  | cause mortality) |  | specified outcome |  |  |  |
| **NRSI** |  |  |  |  |  |  |
| For Partial Yes, must have assessed | | For Yes, must also have assessed RoB: | |  |  |  |
| RoB: |  |  | methods used to ascertain |  | Yes |  |
|  | from confounding, *and* |  | exposures and outcomes, *and* |  | Partial Yes |  |
|  | from selection bias |  | selection of the reported result |  | No |  |
|  |  |  | from among multiple |  | Includes only |  |
|  |  |  | measurements or analyses of a |  | RCTs |  |
|  |  |  | specified outcome |  |  |  |

**10. Did the review authors report on the sources of funding for the studies included in the review?**


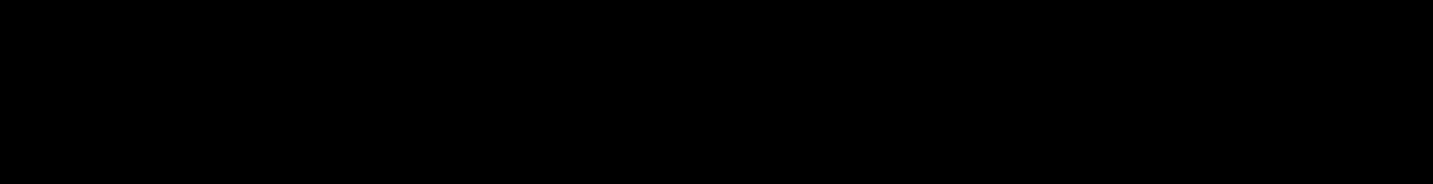

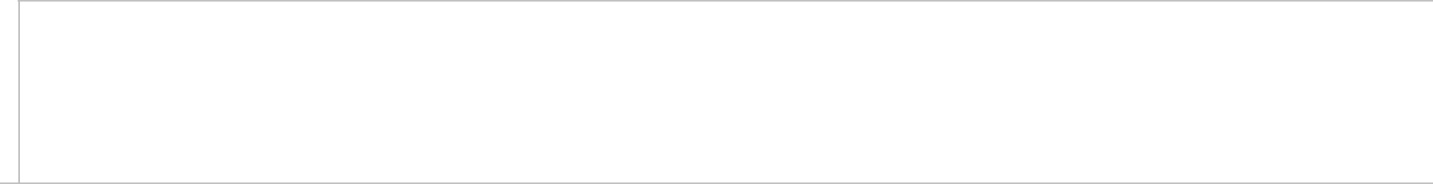


For Yes

- Must have reported on the sources of funding for individual studies included in the review. Note: Reporting that the reviewers looked for this information but it was not reported by study authors also qualifies

1. Yes
2. No
3. **If meta-analysis was performed did the review authors use appropriate methods for statistical combination of results?**

| **RCTs** |  |  |
| --- | --- | --- |
| For Yes: |  |  |
|  The authors justified combining the data in a meta-analysis |  | Yes |
|  AND they used an appropriate weighted technique to combine |  | No |
| study results and adjusted for heterogeneity if present. |  | No meta-analysis |
|  AND investigated the causes of any heterogeneity |  | conducted |
| **For NRSI** |  |  |
| For Yes: |  |  |
|  The authors justified combining the data in a meta-analysis |  | Yes |
|  AND they used an appropriate weighted technique to combine |  | No |
| study results, adjusting for heterogeneity if present |  | No meta-analysis |
|  AND they statistically combined effect estimates from NRSI that |  | conducted |
| were adjusted for confounding, rather than combining raw data, |  |  |
| or justified combining raw data when adjusted effect estimates |  |  |
| were not available |  |  |
|  AND they reported separate summary estimates for RCTs and |  |  |
| NRSI separately when both were included in the review |  |  |

**12. If meta-analysis was performed, did the review authors assess the potential impact of RoB in individual studies on the results of the meta-analysis or other evidence synthesis?**

| For Yes: |  |  |  |
| --- | --- | --- | --- |
|  included only low risk of bias RCTs |  | Yes |  |
|  OR, if the pooled estimate was based on RCTs and/or NRSI at variable |  | No |  |
| RoB, the authors performed analyses to investigate possible impact of |  | No meta-analysis |  |
| RoB on summary estimates of effect. |  | conducted |  |
|  | | |  |
| **13. Did the review authors account for RoB in individual studies when interpreting/ discussing the** | | |  |
| **results of the review?** |  |  |  |
|  |  |  |  |
| For Yes: |  |  |  |
|  included only low risk of bias RCTs |  | Yes |  |
|  OR, if RCTs with moderate or high RoB, or NRSI were included the |  | No |  |
| review provided a discussion of the likely impact of RoB on the results |  |  |  |
|  | | |  |
| **14. Did the review authors provide a satisfactory explanation for, and discussion of, any** | | |  |
| **heterogeneity observed in the results of the review?** |  |  |  |
|  |  |  |  |
| For Yes: |  |  |  |
|  There was no significant heterogeneity in the results |  |  |  |
|  OR if heterogeneity was present the authors performed an investigation of |  | Yes |  |
| sources of any heterogeneity in the results and discussed the impact of this |  | No |  |

on the results of the review

**15.** **If they performed quantitative synthesis did the review authors carry out an adequate investigation of publication bias (small study bias) and discuss its likely impact on the results of the review?**

For Yes:

 performed graphical or statistical tests for publication bias and discussed  Yes

the likelihood and magnitude of impact of publication bias  No

 No meta-analysis conducted

**16. Did the review authors report any potential sources of conflict of interest, including any funding they received for conducting the review?**

| For Yes: | |  |  |
| --- | --- | --- | --- |
|  | The authors reported no competing interests OR |  | Yes |
|  | The authors described their funding sources and how they managed |  | No |
|  | potential conflicts of interest |  |  |

**To cite this tool:** Shea BJ, Reeves BC, Wells G, Thuku M, Hamel C, Moran J, Moher D, Tugwell P,

Welch V, Kristjansson E, Henry DA. AMSTAR 2: a critical appraisal tool for systematic reviews that include randomised or non-randomised studies of healthcare interventions, or both. BMJ. 2017 Sep 21;358:j4008.

| **AMSTAR 2 critical domains (adapted from Shea et al. 2017)** |
| --- |
| •  Protocol registered before commencement of the review (item 2)  •  Adequacy of the literature search (item 4)  •  Justification for excluding individual studies (item 7)  •  Risk of bias from individual studies being included in the review (item 9)  •  Appropriateness of meta-analytical methods (item 11)  •  Consideration of risk of bias when interpreting the results of the review (item 13)  •  Assessment of presence and likely impact of publication bias (item 15)  **Rating overall confidence in the results of the review(adapted from(28)**  **High**  No or one non-critical weakness: the systematic review provides an accurate and comprehensive summary of the results of the available studies that address the question of interest  **Moderate**  More than one non-critical weakness*: the systematic review has more than one weakness but no critical flaws. It may provide an accurate summary of the results of the available studies that were included in the review  **Low**  One critical flaw with or without non-critical weaknesses: the review has a critical flaw and may not provide an accurate and comprehensive summary of the available studies that address the question of interest  **Critically low**  More than one critical flaw with or without non-critical weaknesses: the review has more than one critical flaw and should not be relied on to provide an accurate and comprehensive summary of the available studies  *Multiple non-critical weaknesses may diminish confidence in the review and it may be appropriate to move the overall appraisal down from moderate to low confidence |
