# Supplementary material for: Virtual reality in stroke recovery: a meta-review of systematic reviews
Source: Bioelectron Med. 2024 Oct 5;10:23. doi: 10.1186/s42234-024-00150-9 (PMC11452980; doi:10.1186/s42234-024-00150-9)
Supplement: Supplementary file 5 — Supplementary Material 5. [file 42234_2024_150_MOESM5_ESM.docx]

**Appendix 3**

**Table S3. Excluded references with reason:**

| **Study** | **Exclusion reason** |
| --- | --- |
| (Sisto et al., 2002) | Narrative review |
| (Cameirao et al., 2008) | Narrative review |
| (Penasco-Martin et al., 2010) | Not in English |
| (Laver et al., 2012) | This is outdated, an updated review is included |
| (Poulin et al., 2012) | No VR intervention |
| (Baram. Y, 2013) | This is a narrative review of a VR- technology. Not a systematic review or a meta-analysis |
| (Moreira et al., 2013) | A systematic review but it does not compare VR to CT |
| (Booth et al., 2014) | Mixed conditions |
| (Glegg et al., 2014) | Mixed conditions (a scoping review) |
| (Pietrzah et al., 2014) | Not a systematic review (a scoping review) |
| (Pollock et al., 2014) | General review for interventions UL improvement, VR part covered in a prior included review |
| (Spreij et al., 2014) | No VR stroke related data can be extracted |
| (Turner and Casey, 2014) | No VR stroke related data can be extracted |
| (Crocetta et al., 2015) | Mixed Conditions |
| (Deutsch et al., 2015) | Not a systematic review (a scoping review) |
| (Laver et al., 2015b) | This is outdated, an updated review is included |
| (Nilsen et al., 2015) | General review of interventions post stroke, VR part overlaps with prior included reviews |
| (Darekar et al., 2015) | Not a systematic review (a scoping review) |
| (Ng et al., 2015) | An intervention protocol |
| (Dos Santos et al., 2015) | The studies included did not compare VR with CT |
| (Shin et al., 2015) | The inclusion criteria of this study does not mention comparison with CT |
| (Hatem et al., 2016) | Multiple systematic reviews for general interventions, the stroke part overlaps review already covered |
| (Finestone & Kumbhare, 2016) | This is a second order peer review of previous articles |
| (Hatem et al., 2016) | This was a multiple systematic review focusing both on standard treatment methods and on innovating rehabilitation techniques. It did not compare VR with CT |
| (Yates et al., 2016) | A literature review, not a systematic review |
| (Howard et al., 2017) | This systematic review did not specify patients post-stroke and did not explicitly mention the comparison of VR with CT |
| (Ogourtsova et al., 2017) | This systematic review included more assessment articles than interventional or treatment articles looking at USN neglect post-stroke. Additionally, most of the treatment studies included were not RCTs or systematic reviews. Any systematic reviews that were included in this study were already included in our study |
| (Arienti et al., 2019) | An overview of systematic reviews that did not specifically look into the use of VR compared with CT |
| (Ayed et al., 2019) | A review that did not compare CT with VR for stroke rehabilitation |
| (Dominguez-Tellez et al., 2019) | Not in English |
| (Garcia-Munoz et al., 2019) | Not in English |
| (Lee et al., 2019) | Do not specify a comparison between VR and CT |
| (Lin et al., 2019) | Looked at various rehabilitation measures instead of just VR and CT |
| (Mubin et al., 2019) | Did not specify a comparison with CT |
| (Cassani et al., 2020) | Used a combination of VR with non-invasive brain stimulation |
| (Castellanos-Ruiz e al., 2020) | Not in English |
| (Gandhi et al., 2020) | The study explicated excluded articles containing any form of VR and used only CG |
| (Jung et al., 2020) | Not a systematic review |
| (Kim et al., 2020) | Just a review, not a systematic review |
| (Wu et al., 2020) | Did not explicitly mention how VR compares to CT |
| (Xavier-Rocha et al., 2020) | Restricted search to only Nintendo or Xbox Kinect without any additional VR. Moreover, did not explicitly mention comparison with CT |
| (Chen et al., 2021) | Study focuses on other disorders, minimal number of studies looking at stroke |
| (Freitas et al., 2021) | Did not specifically look at post-stroke impairments |
| (Saeedi et al., 2021) | Used only games with rehabilitation and did not specify a comparison with CT |
| (Voinescu et al., 2021) | Not a systematic review and did not look specifically at stroke population |
| (Wu et al., 2021) | Did not explicitly mention comparison of VR with CT |
| (Fang et al., 2022) | Focused only on VR + CT vs CT |
| (Lv et al., 2022) | Not a systematic review |
| (Patsaki et al., 2022) | Do not explicitly mention the comparison of VR vs CT |
| (Toh et al., 2022) | Do not focus on VR-based rehabilitation |
