# Supplementary material for: Virtual reality in stroke recovery: a meta-review of systematic reviews
Source: Bioelectron Med. 2024 Oct 5;10:23. doi: 10.1186/s42234-024-00150-9 (PMC11452980; doi:10.1186/s42234-024-00150-9)
Supplement: Supplementary file 6 — Supplementary Material 6. [file 42234_2024_150_MOESM6_ESM.docx]

Appendix 4

**Table S4: AMSTAR 2 16-item screening in detail**

| **AMSTAR**  **Study** | **Q**  **1** | **Q**  **2** | **Q**  **3** | **Q**  **4** | **Q**  **5** | **Q**  **6** | **Q**  **7** | **Q**  **8** | **Q**  **9** | **Q**  **10** | **Q**  **11** | **Q**  **12** | **Q**  **13** | **Q**  **14** | **Q**  **15** | **Q**  **16** |
| --- | --- | --- | --- | --- | --- | --- | --- | --- | --- | --- | --- | --- | --- | --- | --- | --- |
| **(36)** | 0 | 0 | 1 | 3 | 1 | 1 | 0 | 3 | 0 | 0 | NA | NA | 0 | 0 | NA | 0 |
| **(37)** | 1 | 3 | 1 | 3 | 1 | 1 | 0 | 3 | 1 | 0 | NA | NA | 1 | 1 | NA | 1 |
| **(30)** | 0 | 3 | 1 | 3 | 1 | 0 | 0 | 0 | 0 | 0 | 1 | 1 | 1 | 1 | 1 | 1 |
| **(38)** | 1 | 3 | 0 | 1 | 1 | 1 | 3 | 1 | 1 | 0 | NA | NA | 1 | 1 | NA | 1 |
| **(39)** | 1 | 3 | 1 | 3 | 1 | 1 | 0 | 1 | 1 | 0 | NA | NA | 1 | 1 | NA | 0 |
| **(19)** | 1 | 3 | 1 | 1 | 1 | 0 | 1 | 1 | 1 | 0 | NA | NA | 1 | 0 | NA | 0 |
| **(18)** | 0 | 3 | 1 | 1 | 1 | 1 | 0 | 1 | 1 | 0 | NA | NA | 1 | 1 | NA | 0 |
| **(40)** | 1 | 3 | 0 | 3 | 1 | 1 | 1 | 1 | 1 | 0 | NA | NA | 0 | 0 | NA | 1 |
| **(41)** | 1 | 1 | 1 | 1 | 1 | 1 | 1 | 1 | 1 | 0 | 1 | 1 | 1 | 1 | 1 | 1 |
| **(42)** | 1 | 3 | 1 | 1 | 1 | 1 | 0 | 1 | 1 | 0 | 1 | 1 | 1 | 1 | 0 | 1 |
| **(43)** | 0 | 0 | 1 | 3 | 1 | 1 | 0 | 1 | 1 | 0 | NA | NA | 1 | 1 | NA | 0 |
| **(44)** | 1 | 3 | 1 | 1 | 0 | 0 | 0 | 1 | 1 | 0 | NA | NA | 1 | 0 | NA | 1 |
| **(45)** | 1 | 3 | 1 | 1 | 1 | 1 | 1 | 1 | 1 | 0 | 1 | 1 | 1 | 1 | 0 | 0 |
| **(23)** | 1 | 3 | 1 | 1 | 1 | 1 | 0 | 1 | 1 | 0 | 1 | 1 | 1 | 1 | 1 | 1 |
| **(46)** | 1 | 3 | 1 | 3 | 1 | 0 | 0 | 1 | 1 | 0 | NA | NA | 1 | 1 | NA | 1 |
| **(47)** | 1 | 3 | 1 | 1 | 1 | 1 | 1 | 1 | 1 | 0 | 1 | 1 | 1 | 1 | 1 | 0 |
| **(48)** | 1 | 1 | 1 | 3 | 1 | 1 | 1 | 1 | 1 | 0 | 1 | 1 | 1 | 1 | 0 | 1 |
| **(49)** | 0 | 0 | 0 | 3 | 1 | 0 | 1 | 1 | 1 | 0 | 1 | 1 | 1 | 1 | 0 | 3 |
| **(50)** | 1 | 3 | 1 | 3 | 1 | 0 | 0 | 3 | 1 | 0 | NA | NA | 1 | 1 | NA | 1 |
| **(51)** | 1 | 0 | 1 | 1 | 1 | 1 | 3 | 1 | 1 | 0 | 3 | 0 | 0 | 3 | 0 | 1 |
| **(2)** | 1 | 3 | 1 | 1 | 1 | 1 | 3 | 1 | 1 | 0 | 1 | 1 | 1 | 1 | 1 | 1 |
| **(20)** | 1 | 1 | 1 | 1 | 1 | 1 | 1 | 1 | 1 | 0 | 1 | 1 | 1 | 1 | 1 | 1 |
| **(52)** | 1 | 3 | 1 | 3 | 1 | 1 | 3 | 1 | 1 | 0 | 1 | 1 | 1 | 0 | 3 | 1 |
| **(53)** | 1 | 0 | 1 | 1 | 1 | 1 | 3 | 1 | 3 | 0 | 1 | 1 | 1 | 1 | 1 | 1 |
| **(54)** | 3 | 0 | 1 | 3 | 1 | 0 | 3 | 1 | 0 | 0 | NA | NA | 0 | 0 | NA | 1 |
| **(55)** | 1 | 1 | 1 | 1 | 3 | 1 | 1 | 1 | 1 | 0 | 1 | 3 | 1 | 1 | 0 | 0 |
| **(56)** | 1 | 0 | 1 | 3 | 1 | 0 | 3 | 1 | 1 | 0 | 1 | 1 | 1 | 1 | 0 | 1 |
| **(57)** | 1 | 0 | 3 | 3 | 1 | 1 | 3 | 1 | 1 | 0 | 1 | 1 | 1 | 1 | 1 | 1 |
| **(58)** | 1 | 0 | 3 | 3 | 1 | 1 | 3 | 1 | 1 | 0 | 1 | 3 | 0 | 0 | 0 | 1 |
| **(59)** | 1 | 3 | 1 | 3 | 1 | 0 | 3 | 1 | 1 | 0 | 1 | 1 | 1 | 1 | 0 | 1 |
| **(60)** | 1 | 0 | 1 | 3 | 1 | 1 | 0 | 3 | 1 | 0 | 1 | 0 | 1 | 0 | 0 | 1 |
| **(61)** | 1 | 0 | 3 | 3 | 1 | 1 | 0 | 1 | 1 | 0 | NA | NA | 1 | 0 | NA | 1 |
| **(62)** | 1 | 0 | 0 | 1 | 1 | 1 | 0 | 1 | 1 | 0 | 1 | 1 | 1 | 1 | 0 | 1 |
| **(63)** | 1 | 1 | 3 | 1 | 1 | 1 | 0 | 1 | 1 | 0 | 1 | 1 | 1 | 1 | 1 | 1 |
| **(64)** | 1 | 1 | 1 | 3 | 1 | 1 | 3 | 1 | 1 | 0 | 1 | 1 | 1 | 1 | 1 | 1 |
| **(65)** | 1 | 0 | 1 | 1 | 1 | 1 | 0 | 1 | 1 | 0 | 1 | 3 | 1 | 3 | 0 | 1 |
| **(66)** | 1 | 1 | 3 | 3 | 1 | 1 | 3 | 1 | 1 | 0 | 1 | 1 | 3 | 0 | 1 | 1 |
| **(67)** | 1 | 0 | 3 | 1 | 1 | 1 | 0 | 1 | 1 | 0 | 1 | 1 | 1 | 0 | 0 | 1 |
| **(68)** | 1 | 0 | 1 | 1 | 1 | 1 | 3 | 1 | 1 | 0 | 1 | 0 | 3 | 1 | 1 | 1 |
| **(69)** | 1 | 0 | 3 | 1 | 1 | 1 | 3 | 1 | 1 | 0 | 1 | 1 | 1 | 1 | 1 | 1 |
| **(70)** | 1 | 1 | 3 | 3 | 1 | 1 | 3 | 1 | 1 | 0 | 1 | 1 | 1 | 1 | 0 | 1 |
| **(71)** | 1 | 1 | 3 | 1 | 1 | 1 | 0 | 1 | 1 | 0 | 1 | 1 | 1 | 1 | 1 | 1 |
| **(72)** | 1 | 0 | 1 | 3 | 1 | 1 | 3 | 1 | 1 | 0 | NA | NA | 1 | 3 | NA | 1 |
| **(73)** | 1 | 0 | 1 | 1 | 1 | 1 | 1 | 1 | 1 | 0 | 1 | 1 | 1 | 1 | 1 | 1 |
| **(74)** | 1 | 0 | 3 | 1 | 1 | 1 | 1 | 1 | 1 | 0 | 1 | 1 | 3 | 3 | 1 | 1 |
| **(75)** | 1 | 1 | 3 | 1 | 1 | 1 | 1 | 1 | 1 | 0 | 1 | 1 | 1 | 1 | 1 | 1 |
| **(76)** | 1 | 0 | 1 | 3 | 1 | 1 | 0 | 1 | 1 | 0 | 1 | 1 | 1 | 1 | 1 | 1 |
| **(77)** | 1 | 1 | 3 | 3 | 1 | 1 | 1 | 1 | 1 | 0 | 1 | 1 | 3 | 1 | 1 | 1 |
| **(78)** | 1 | 1 | 3 | 3 | 1 | 1 | 1 | 1 | 1 | 0 | 1 | 3 | 3 | 3 | 0 | 1 |
| **(79)** | 1 | 1 | 3 | 3 | 1 | 1 | 1 | 1 | 1 | 0 | 1 | 1 | 3 | 3 | 0 | 1 |
| **(80)** | 1 | 0 | 3 | 1 | 1 | 1 | 0 | 1 | 1 | 0 | 1 | 3 | 0 | 3 | 1 | 1 |
| **(81)** | 1 | 0 | 3 | 1 | 1 | 1 | 0 | 1 | 1 | 0 | 1 | 1 | 1 | 1 | 1 | 1 |
| **(82)** | 1 | 1 | 3 | 1 | 1 | 1 | 1 | 1 | 1 | 0 | 1 | 1 | 1 | 1 | 0 | 1 |
| **(33)** | 1 | 1 | 1 | 3 | 1 | 1 | 1 | 1 | 1 | 0 | NA | NA | 1 | 3 | NA | 1 |
| **(83)** | 1 | 0 | 3 | 1 | 3 | 3 | 3 | 1 | 1 | 0 | NA | NA | 1 | 1 | NA | 1 |
| **(84)** | 1 | 0 | 3 | 3 | 1 | 1 | 3 | 1 | 1 | 0 | 1 | 1 | 1 | 3 | 0 | 1 |
| **(32)** | 1 | 0 | 3 | 1 | 1 | 1 | 1 | 1 | 1 | 0 | 1 | 1 | 1 | 3 | 0 | 1 |

0=No,1=yes, 3= Partial yes, NA=Not Applicable
